# A comprehensive fate map by intracellular injection of identified blastomeres in the marine polychaete *Capitella teleta*

**DOI:** 10.1186/2041-9139-1-8

**Published:** 2010-09-15

**Authors:** Néva P Meyer, Michael J Boyle, Mark Q Martindale, Elaine C Seaver

**Affiliations:** 1 Kewalo Marine Laboratory, Pacific Biosciences Research Center, University of Hawaii, 41 Ahui Street, Honolulu, Hawaii 96813, USA; 2 Smithsonian Marine Station at Fort Pierce, 701 Seaway Drive, Fort Pierce, Florida 34949, USA

## Abstract

**Background:**

The polychaete annelid *Capitella teleta *(formerly *Capitella *sp. I) develops by spiral cleavage and has been the focus of several recent developmental studies aided by a fully sequenced genome. Fate mapping in polychaetes has lagged behind other spiralian taxa, because of technical limitations.

**Results:**

To generate a modern fate map for *C. teleta*, we injected 1,1'-dioctadecyl-3,3,3'3'-tetramethylindocarbocyanine perchlorate (DiI) into individual identified blastomeres through fourth-quartet micromere formation. Confocal laser scanning microscopy at single-cell resolution was used to characterize blastomere fates during larval stages. Our results corroborate previous observations from classic studies, and show a number of similarities with other spiralian fate maps, including unique and stereotypic fates for individual blastomeres, presence of four discrete body domains arising from the A, B, C and D cell quadrants, generation of anterior ectoderm from first quartet micromeres, and contributions to trunk ectoderm and ventral nerve cord by the 2d somatoblast. Of particular interest are several instances in which the *C. teleta *fate map deviates from other spiralian fate maps. For example, we identified four to seven distinct origins of mesoderm, all ectomesodermal. In addition, the left and right mesodermal bands arise from 3d and 3c, respectively, whereas 4d generates a small number of trunk muscle cells, the primordial germ cells and the anus. We identified a complex set of blastomere contributions to the posterior gut in *C. teleta*, which establishes the most complete map of posterior gut territories to date.

**Conclusions:**

Our detailed cellular descriptions reveal previously underappreciated complexity in the ontogenetic contributions to several spiralian larval tissues, including the mesoderm, nervous system and gut. The formation of the mesodermal bands by 3c and 3d is in stark contrast to other spiralians, in which 4d generates the mesodermal bands. The results of this study provide a framework for future phylogenetic comparisons and functional analyses of cell-fate specification.

## Background

Many metazoan embryos develop via highly stereotyped cleavage programs that enable the identification of individual blastomeres during early development. Embryonic features that aid identification include differences in cell size or pigmentation, or in spindle orientation relative to the primary egg axis. Such embryos are amenable to cell-lineage and fate-mapping studies, which establish the developmental origins of definitive regions, tissues and organs in larval and adult animals, and provide the groundwork for functional studies. Cell-lineage and fate-mapping studies were among the first rigorous attempts at characterizing embryogenesis in the late 19th century in embryos as diverse as parasitic nematodes, ascidians, ctenophores, annelids, mollusks and various other marine invertebrates. The ability to follow descendants of identified cells has increased dramatically in recent years with the advent of improved fluorescent reagents for intracellular labeling and advanced imaging techniques.

Early cell-lineage and fate-mapping studies revealed that animals with dissimilar adult body plans probably shared a common evolutionary ancestor, based on similar developmental features. A spectacular example is a group of animals that displays a pattern of early development called spiral cleavage. This pattern of development is recognizable by the timing, orientation and/or size of individual cell divisions, and is found in a large number of diverse animal groups including mollusks, annelids, sipunculans, echiurans, nemerteans, myzostomids, ectoprocts, polyclad flatworms and potentially gnathostomulids. Molecular phylogenomic analyses [1-4] have indicated that the spiral cleavage program was probably an ancestral characteristic of all non-ecdysozoan protostomes (lophotrochozoans) that was subsequently lost in select taxa including lophophorates (brachiopods, phoronids), gastrotrichs, rotifers, parasitic (non-polyclad) platyhelminthes and cephalopod mollusks. However, a better understanding of the exact relationships among lophotrochozoans, particularly between members of the Platyzoa (for example, gnathostomulids, gastrotrichs, rotifers and platyhelminthes), is needed to determine whether spiral cleavage was an ancestral character for all lophotrochozoans (Spiralia) or just a subset (Trochozoa).

**Figure 1:**
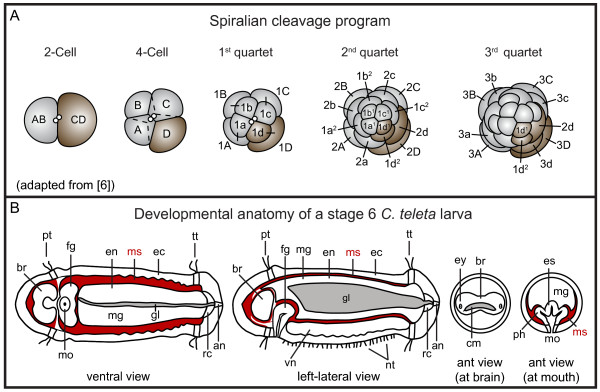
**Spiral cleavage and *Capitella teleta *larval body plan**. (**A**) Diagram of unequal spiral cleavage. (**B**) Diagram of a late stage 6/early stage 7 *Capitella teleta *larva. Mesoderm is shown in red. an = anus, br = brain, cm = commissure, ec = ectoderm, en = endoderm, es = esophagus, ey = eye, fg = foregut, gl = gut lumen, mg = midgut, mo = mouth, ms = mesoderm, nt = neurotroch, ph = pharynx, pt = prototroch, rc = rectum, tt = telotroch, vn = ventral nerve cord.

During spiral cleavage, the cleavage spindles of the first two divisions are oriented perpendicular to the animal-vegetal axis, and divide the zygote into four quadrants. The cells born from these first divisions are denoted the A, B, C and D blastomeres (Figure 1A). In animals with unequal spiral cleavage, the first two divisions are unequal in size, allowing for unambiguous identification of each blastomere. Starting with the third cleavage, the four macromeres generate tiers of often smaller, animal daughters (micromeres) in alternating orientation (Figure 1A). In the majority of spiralians, the first tier of micromeres is cleaved in a clockwise (dexiotropic) direction when viewed from the animal pole. This is followed by a counterclockwise cleavage (laeotropic) of the second tier of micromeres. These divisions result from the alternating 90 degree orientation of the mitotic spindles, and produce a 'spiral' pattern of micromeres, the hallmark of the spiral cleavage program. Blastomere nomenclature follows that of Conklin [5]. Macromeres are denoted by an upper case letter and micromeres by a lower case letter, corresponding to their quadrant of origin (Figure 1A). Each blastomere has a number corresponding to its birth order. For example, after the first spiral cleavage (eight-cell stage), the macromeres are named 1A, 1B, 1C and 1D, and the micromeres are named 1a, 1b, 1c and 1d; after the second spiral cleavage (16-cell stage), the macromeres are 2A, 2B, 2C and 2D and the micromeres are 2a, 2b, 2c and 2d (Figure 1A). Subsequent micromere divisions are denoted by numerical superscripts, a '1' for the animal-most daughter and a '2' for the vegetal daughter. For example, first quartet micromeres divide to give rise to four animal micromere cells (1a^1^, 1b^1^, 1c^1 ^and 1d^1^) and four vegetal micromere cells (1a^2^, 1b^2^, 1c^2 ^and 1d^2^).

In addition to the conserved pattern of spiral cleavage, blastomere fates are also largely conserved. Generally, animal micromeres give rise to ectoderm, whereas vegetal macromeres give rise to endoderm. Another frequently conserved fate is that of the left and right larval eyes, which are generated by micromeres 1a and 1c, respectively. Of particular importance in spiralian development are two cells derived from the D quadrant. In annelids, the 2d micromere, called the primary somatoblast, gives rise to the majority of trunk ectoderm posterior to the mouth. The 4d cell, called the mesentoblast, is the only micromere in any spiralian embryo that generates both mesoderm and endoderm [6,7].

Although early descriptive work on spiralian embryos emphasized similarities in the fates of identified cells, modern intracellular studies have identified a number of species-specific differences. For example, the larval eyes of chitons (polyplacophoran mollusks) are generated from the second quartet micromeres 2a and 2c, rather than from 1a and 1c as observed in all other species examined [8]. Likewise, the origin of ectomesoderm, which is mesoderm derived from the first three quartets of ectodermal micromeres, varies across species and contrasts with the highly conserved origin of endomesoderm derived from 4d [7,9-11]. Differences in blastomere fate among species probably reflect meaningful phylogenetic variation in the development of homologous cells over evolutionary time, and provide a foundation for molecular investigations of the causal basis of cell-fate determination.

Polychaete annelids are a widely diverse group of primarily marine segmented worms. Although there are classic cell-lineage descriptions from the late 19th century, generation of fate maps for polychaetes using intracellular lineage tracers has lagged behind those of other taxa. The only published intracellular fate-mapping study of a polychaete annelid is for the ragworm *Platynereis dumerilii*, in which the first quartet micromeres (1a to 1d) and macromeres (1A to 1D), 2d^112^, 4d and 4d^1 ^were directly filled [12]. Although there are substantial detailed lineage data for clitellid annelids (leeches and oligochetes), these animals have a modified spiral cleavage program that gives rise to a specialized set of ectodermal and mesodermal teloblast cells not described in polychaetes. To generate a modern fate map for the polychaete annelid *Capitella teleta *[13], previously known as *Capitella *sp. I, we injected the fluorescent dye 1,1'-dioctadecyl-3,3,3'3'-tetramethylindocarbocyanine perchlorate (DiI) intracellularly into identified blastomeres, and examined their fate at larval stages using confocal laser scanning microscopy. We determined the fates of each blastomere through formation of the fourth quartet of micromeres. The results of this study are compared with fate maps of other spiralian embryos, with particular emphasis on those fate maps generated using intracellular injections.

## Results

### *C. teleta *development and fate map overview

*C. teleta *embryos develop by unequal spiral cleavage, thus individual blastomeres are easily identifiable. Starting at the two-cell stage, divisions occur approximately every hour and are roughly synchronous between quadrants, although the D quadrant generally begins dividing first. At the four-cell stage, the D macromere is the largest cell, and shares a vegetal cross furrow with B. In this paper, we use a lower case 'q' to refer to each micromere quartet and an upper case 'Q' to refer to each macromere quartet. The first quartet micromeres (1q) are born dexiotropically (clockwise) with respect to the macromeres when viewed from the animal pole; second quartet micromeres (2q) are born laeotropically (counterclockwise), and subsequent macromere cleavages alternate between dexiotropic and laeotropic. Our observations of the early cleavages of *C. teleta *are very similar to the descriptions of *Capitella capitata *cleavages by Eisig in 1898 [14]. One notable exception is the size of 4d: Eisig describes 4d as much larger than other fourth quartet micromeres, whereas in *C. teleta*, we found 4d to be the same size as other 4q cells.

A standard embryonic and larval staging system has been described previously for *C. teleta *[15]. In general, after 5 days of development at 19°C, the majority of larval and adult structures are discernable. At this stage (late stage 6, early stage 7), the larva consists of an anterior head region, a segmented trunk and a posterior pygidium. The trunk is bounded by two ciliary bands: the prototroch (pt) anteriorly, and the telotroch (tt) posteriorly (Figure 1B). A third ciliary band, the neurotroch (nt), runs along the ventral midline (Figure 1B). There are also rows of cilia in the pygidium called the pygidial ciliary band (not shown in diagram) [16]. The larva has a centralized nervous system consisting of an anterior brain or cerebral ganglion (br) and a ventral nerve cord (vn) consisting of up to 13 segmentally reiterated ganglia (Figure 1B). The cerebral commissure (cm) and pair of larval eyes (ey) are also visible (Figure 1B). The mesoderm (ms) is positioned between the ectoderm (ec) and endoderm (en) (Figure 1B), and many differentiated circular and longitudinal muscle fibers are present by this stage. The gut is regionalized along the anterioposterior axis into a foregut (fg), midgut (mg) and hindgut. The foregut is further subdivided into a buccal cavity, pharynx (ph) and esophagus (es), and we used the term 'mouth' (mo) to refer to the cells lining the opening of the buccal cavity (Figure 1B). At mid to late larval stages, the mouth is continuous with the presumptive pharynx and esophagus [17]The midgut in *C teleta *comprises an intestine that extends from the esophagus to the rectum. Traditionally, the 'hindgut' in polychaetes is described as a proctodeal invagination of ectoderm [18,19]. To more accurately interpret and compare the *C. teleta *fate map with other spiralian fate maps, we used the terms 'rectum' and 'anus' when referring to the posteriormost end (hindgut) of the alimentary canal. In the larva of *C. teleta*, the rectum (rc) is a short region in the pygidium that connects the intestine with a terminal anus (an) (Figure 1B). By late stage 6, a lumen (gl) is visible within the developing midgut and rectum (Figure 1B). Late stage 6 *C. teleta *larvae are competent to metamorphose after another 3 to 4 days of development at 19ĀC (stage 9).

**Figure 2:**
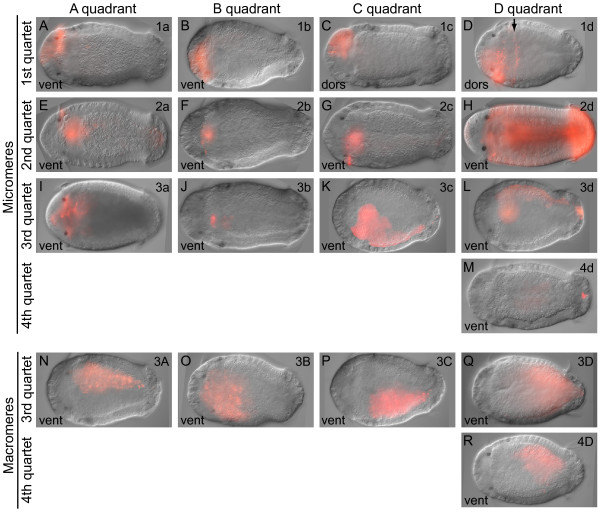
**General fate map of *Capitella teleta***. (**A-M**) Stage 6 to 7 larvae ~5 days after labeling the first to fourth quartet micromeres. (**N-R**) Stage 6 to 7 larvae ~5 days after labeling the third and fourth quartet macromeres. In all panels, differential interference contrast (DIC) images are overlaid with red DiI fluorescent images. All images are of fixed larvae except for **(D) **and **(H) **and **(I)**, which are of live animals. The arrow in **(D) **indicates a row of DiI-labeled ectodermal cells posterior to the prototroch. The blastomere labeled with DiI is indicated in the upper-right corner, and the view is indicated in the lower-left corner of each panel (dors = dorsal, vent = ventral). Anterior is to the left in all panels.

The injection of individual blastomeres resulted in clones of labeled descendant cells that were highly reproducible, enabling us to generate a stereotypic fate map for *C. teleta*. Blastomeres 1q, 1q^1^, 1q^2^, 2q, 3q, 4d, 2Q, 3Q and 4D were injected with DiI (Table 1), allowed to develop to stages 5 to 8, and scored as alive or fixed. Most animals were scored between stages 6 and 7 because visualization of DiI at later stages (8 and 9) is difficult in large clones because of the dilution of DiI. In brief, first quartet micromeres generate the anterior ectoderm including the brain and prototroch (Figure 2A-D). The second quartet micromeres generate the ectoderm posterior to the prototroch, the ventral nerve cord, portions of the mouth, the majority of the foregut, a single posterior row of prototrochal cells, the telotroch and rectum (Figure 2E-H). The third quartet of micromeres give rise to portions of the foregut and mouth, anterior mesoderm, cells surrounding the anus, and the left and right mesodermal bands in the trunk (Figure 2I-L). 4d forms a few muscle cells, the anus and the primordial germ cells (Figure 2M). Finally, macromeres 3A, 3B, 3C and 4D generate endoderm (Figure 2N-R). This fate map is largely consistent with the fate map of other spiralians, especially with respect to the ectodermal fates. The main deviation is that 4d does not generate the mesodermal bands; this fate is divided between 3c and 3d.

**Table 1:** Number of larvae scored after injection of individual identified blastomeres.

	Micromeres				Macromeres			
	
	a	b	c	d	A	B	C	D
First quartet	12	15	13	18	-	-	-	-

Second quartet	17	12	27	22	2	4	17	18

Third quartet	18	14	22	22	13	10	9	20

Fourth quartet	-	-	-	23	-	-	-	11

### First quartet micromeres

**Figure 3:**
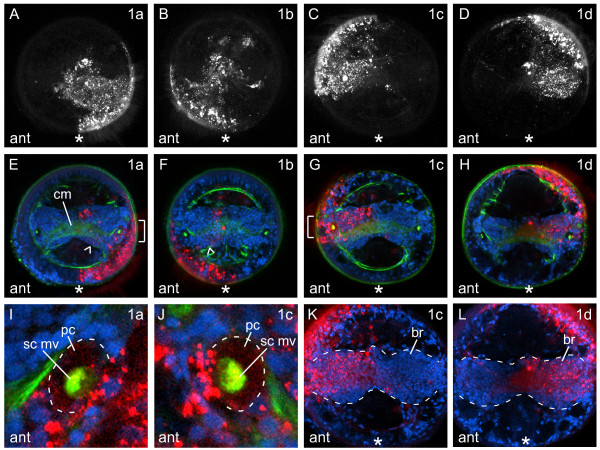
**First quartet micromeres generate anterior ectoderm and prototroch**. (**A-D**) Single-channel, z-stacks of confocal images of late stage 6 larvae from an anterior view 5 days after labeling first quartet micromeres with DiI (white). The stacks start at the anterior ectodermal surface and end just posterior to the prototroch. (**E-H**) Z-stacks of merged, confocal images through a subset of the brain in late stage 6 larvae. The channels are DiI (red), phalloidin (green) and TO-PRO-3 (blue). **(E, F) **Labeled brain cells positioned ventral to the cerebral commissure are indicated with an arrowhead in **(E,F)**. (**I-J**) Digital magnification of the eye region bracketed in **(E) **and **(G)**, respectively. One side of the eye sensory cell is outlined with a dashed line. (**K-L**) Z-stacks of merged, confocal images through the entire brain of late stage 6 larvae. The channels are DiI (red) and TO-PRO-3 (blue). The blastomere labeled with DiI is indicated in the upper-right corner, and the view is indicated in the lower-left corner of each panel (ant = anterior). All images are from an anterior view, with ventral down. The position of the mouth is indicated with an asterisk. br = brain, cm = brain commissure, pc = pigment cell, sc mv = sensory cell microvilli.

In *C. teleta*, descendents of the first quartet micromeres are subdivided between the left-right and dorsal-ventral quadrants of the anterior ectoderm (1a, left-ventral; 1b, right-ventral; 1c, right-dorsal; 1d, left-dorsal) (Figure 3A-D). Micromeres 1a to 1d give rise to the anterior ectoderm, brain (or cerebral ganglion), larval eyes and the prototroch (Figure 3).

#### Micromere 1a

Descendants of the 1a micromere form left-ventral head ectoderm and the left-ventral prototroch (Figure 3A, E). In *C. teleta*, there are two larval eyes, each consisting of three cells: a sensory cell, a pigment cell and a supporting cell [20]. Micromere 1a clearly forms the pigment cell (pc) and sensory cell of the left eye, including the microvilli of the sensory apparatus (sc mv), which are visible with phalloidin staining (Figure 3I). Micromere 1a also probably generates the supporting cell of the left eye, although this is more difficult to determine. In addition to the left-ventral head ectoderm, left-ventral prototroch and left eye, a small number of cells in the left side of the brain, ventral to the cerebral commissure (cm), are descendants of 1a (Figure 3E, arrowhead).

#### Micromere 1b

The 1b micromere generates right-ventral head ectoderm, the right-ventral prototroch and a small number of cells in the right-ventral brain (arrowhead) (Figure 3B, F). This pattern largely mirrors that of 1a descendants, with the exception of the left eye (compare Figure 3E with Figure 3F).

#### Micromere 1c

Micromere 1c gives rise to right-dorsal head ectoderm, the right-dorsal prototroch (Figure 3C, G) and the right eye (Figure 3J). Similar to the 1a micromere, the 1c micromere forms the pigment cell, the sensory cell and probably the supporting cell of the right eye (Figure 3J). Micromere 1c also forms the majority of the right side of the brain (br) (Figure 3G, K). DiI-labeled cells in the brain are often positioned dorsal to the cerebral commissure, and DiI is seen in the cerebral commissure (Figure 3G). Descendants of 1c are also found in ectoderm just posterior to the prototroch, both on the dorsal midline and on the dorsolateral sides of the larva (not shown). Some of these cells may be sensory neurons.

#### Micromere 1d

The 1d micromere generates left-dorsal head ectoderm, the left-dorsal prototroch (Figure 3D, H) and the majority of the left side of the brain (br) (Figure 3L). DiI-labeled cells in the brain are usually positioned dorsal to the cerebral commissure, and DiI is seen in the cerebral commissure (Figure 3H). Descendants of 1d also give rise to a thin line of ectodermal cells and scattered surface cells in the trunk. The line of ectodermal cells forms a ring that partially encircles the larva, terminating on the ventral face, just lateral to the mouth. This ring of 1d-derived cells is positioned posterior to the mouth, between two rows of pigment cells (Figure 2D, arrow). The scattered trunk ectodermal cells formed by 1d are localized to the dorsal and dorsolateral sides of the larva, and are positioned between the prototroch and line of 1d ectodermal cells (not shown). Some of these cells are sensory neurons, whereas others have a distinct 'S'-shaped morphology. Phalloidin staining of the 'S'-shaped cells shows repeated actin rings along the outside of each cell. Cells with this 'S'-shaped morphology are found throughout the surface ectoderm in the head, trunk and pygidium. The pattern generated by micromere 1d largely mirrors that seen after labeling 1c, with the exception of the right eye and ectodermal cells in the trunk (compare Figure 3G and 3K with Figure 3H and 3L).

#### Micromeres 1q^1 ^and 1q^2^

**Figure 4:**
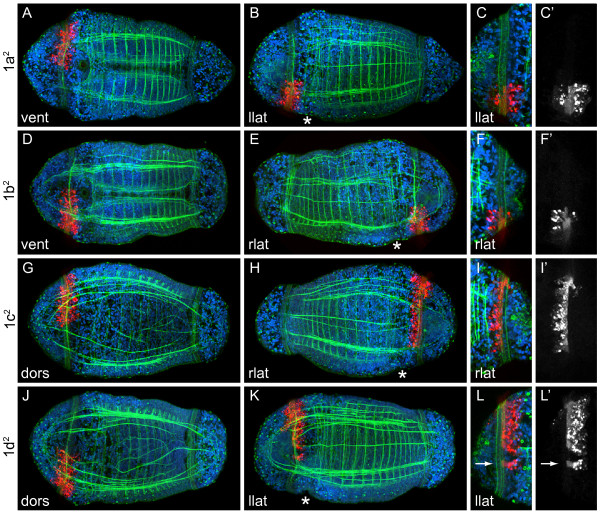
**Vegetal daughters of first quartet micromeres, 1q^2^, form the prototroch**. (**A, B, D, E, G, H, J, K**) Z-stacks of merged, confocal images through late stage 6 larvae 5 days after labeling 1q^2 ^micromeres with DiI. The channels are DiI (red), phalloidin (green) and TO-PRO-3 (blue). (**C, F, I, L**) Cropped, digitally magnified, z-stacks of merged, confocal images through the prototroch of late stage 6 larvae. The channels are DiI (red), phalloidin (green) and TO-PRO-3 (blue). (**C', F', I', L'**) Single channel images of DiI shown in **(C), (F), (I) **and **(L)**, respectively. The arrow in **(L) **points to a DiI-labeled prototroch cell that is separate from the rest of the clone. The blastomere labeled with DiI is indicated to the left of each row and the view is indicated in the lower-left corner of each panel (dors = dorsal, vent = ventral, llat = left lateral, rlat = right lateral). Anterior is to the left in all ventral, dorsal and left lateral images, and to the right in all right lateral images. The position of the mouth is indicated with an asterisk.

**Figure 5:**
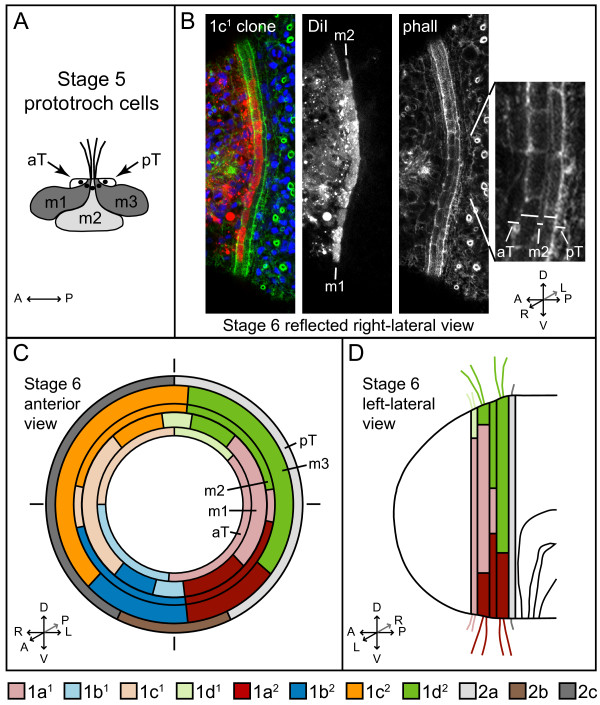
**Micromere contributions to the prototroch in *Capitella teleta***. (**A**) Diagram drawn from a confocal slice through a stage 5 larva showing five prototrochal cells. At this stage, each of the main rows of prototochal cells (m1, m2, m3) meets apically to form the densely ciliated region of the prototroch. The nuclei (black circles) are positioned apically for all five prototrochal cells. (**B**) Cropped z-stack of merged (left image) or single-channel (channel indicated in the top left corner of each image), confocal images through the prototroch of a late stage 6 larva after injecting 1c^1 ^with DiI. The images are a reflected right lateral view. The channels in the merged image are DiI (red), phalloidin (green) and TO-PRO-3 (blue). The image to the far-right is a close-up view of the single-channel phalloidin image. Each row of the prototroch is indicated with a horizontal line. (**C, D**) Anterior and left lateral diagrams of a late stage 6 larva showing micromere contributions to the five rows of prototrochal cells. Descendants of each micromere are indicated by specific colors, which are shown below the diagrams (light red = 1a^1^, light blue = 1b^1^, light orange = 1c^1^, light green = 1d^1^, red = 1a^2^, blue = 1b^2^, orange = 1c^2^, green = 1d^2^, light grey = 2a, brown = 2b, dark grey = 2c). The orientation of the diagrams or confocal images is indicated with arrows. A = anterior, aT = accessory prototroch, D = dorsal, L = left, m = main prototroch, P = posterior, phall = phalloidin, pT = posterior accessory prototroch, R = right, V = ventral.

The vegetal daughters of the first quartet micromeres, 1a^2 ^to 1d^2 ^(Figure 4), were labeled because in many other spiralians, these cells generate most of the prototroch. We examined clones generated by 1q^2 ^micromeres both at late stage 6 and earlier at stage 5 when the prototochal cells are larger and cell boundaries are easier to discern. In *C. teleta*, we detected five rows of prototrochal cells (Figure 5A). At late stage 6, the second and fourth rows of prototrochal cells are densely ciliated, whereas the first and fifth rows are more sparsely ciliated. The third row may also be densely ciliated, but we could not determine this at late stage 6. At late stage 4 and early stage 5, cells in the second, third and fourth rows are relatively large compared with cells in the first and fifth rows (Figure 5A). Because of the pattern of ciliation and the size of the cells in each row, we refer to the second through fourth rows as the main prototroch, numbered 1 to 3 from anterior to posterior (m1, m2, m3, respectively). We refer to the first row as the anterior supporting prototroch (aT) and the fifth row as the posterior supporting prototroch (pT).

The 1q^2 ^micromeres of *C. teleta *generate the majority of the densely ciliated main prototroch, including all of the fourth row (m3), most of the third row (m2) and a subset of the second row (m1) of prototrochal cells (Figure 4; Figure 5C,D). In general, 1a^2 ^forms the left-ventral region (n = 11; Figure 4A-C'), 1b^2 ^forms the right-ventral region (n = 15; Figure 4D-F'), 1c^2 ^forms the right-dorsal region (n = 13; Figure 4G-I') and 1d^2 ^forms the left-dorsal region (n = 14; Figure 4J-L'). The main exception to this occurs laterally. Clones from 1c^2 ^and 1d^2 ^generate a larger circumferential area than do those from 1a^2 ^and 1b^2 ^(compare Figure 4H and 4K with 4B and 4E; Figure 5C,D). Additionally, on the lateral sides of the prototroch, 1c^2 ^and 1d^2 ^only contribute to m2 and m3 (Figure 4H-I', K-L'; Figure 5C,D).

We also labeled the 1q^1 ^blastomeres (n = 5 for each micromere) to determine whether these cells contribute to the prototroch. In general, descendants of 1q^1 ^form the entire anterior supporting prototroch (1a^1^, left-ventral; 1b^1^, right-ventral; 1c^1^, right-dorsal; 1d^1^, left-dorsal) and part of the main prototroch, including rows m1 (1a^1^, left; 1b^1^, ventral; 1c^1^, right; 1d^1^, dorsal) and m2 (1a^1^, left; 1c^1^, right) (Figure 5C,D). The left and right lateral sides of row m1 are generated by 1a^1 ^and 1c^1^, respectively (Figure 5B-D), which also generate the left and right eyes. 1b^1 ^and 1d^1 ^contribute a few daughters to the ventral and dorsal regions of the m1 prototroch row (Figure 5C). 1q^1 ^blastomeres also generate the anterior ectoderm, the brain, a thin ring of body ectoderm, and the 'S'-shaped cells and sensory neurons in the trunk.

Interestingly, the boundaries of each clone within the prototroch are not completely stereotyped. In the larvae examined, we found intercalation between clones and clonal boundaries that were shifted with respect to the animal axes from animal to animal. One, two or no DiI-labeled prototrochal cells were found to be interspersed with unlabeled cells, and the number of labeled cells separate from the bulk of the clones was not the same for all animals. For example, the animal in Figure 4L has a single prototrochal cell separated from the rest of the clone (arrow); however, in other animals in which 1d^2 ^was labeled, no cells, several single cells, or two cells were found separated from the bulk of the clone (not shown). This mixing at clonal boundaries may result from the large number of prototrochal cells generated in *C. teleta *versus other spiralians in which the prototrochal cells become mitotically arrested early in development [21]. In *C. teleta*, the second (m1) and fourth (m3) rows of prototrochal cells each contained ~55 cells at late stage 6 (five animals scored: 50, 54, 54, 58 and 60 m1 cells). Cells in the third row (m2) were more difficult to count precisely, but there appeared to be ~1 cell for every 1.4 cells in the second row (~ 39 m2 cells). We also found that prototrochal clones were somewhat variable in cell number, although this could be due to developmental differences. For example, 1b^1 ^contributed four or six cells to the m1 prototrochal row at late stage 6.

### Second quartet micromeres

**Figure 6:**
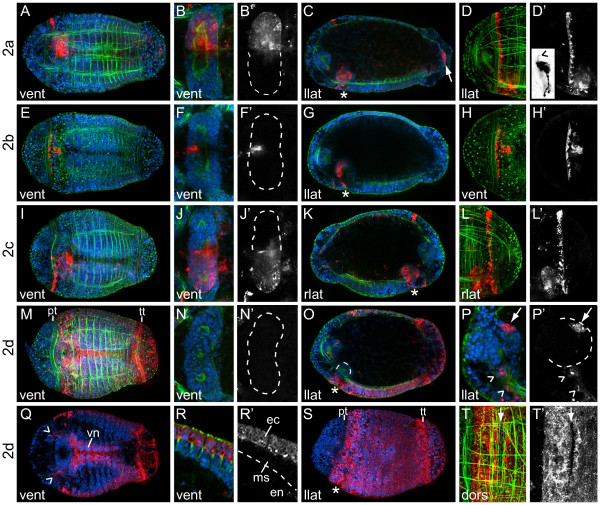
**Second quartet micromeres generate foregut and trunk ectoderm**. (**A-T**) Z-stacks of merged, confocal images of late stage 6 larvae 5 days after labeling second quartet micromeres with DiI. The channels are DiI (red), phalloidin (green) and TO-PRO-3 (blue) except for **(D)**, **(H)**, **(L) **and **(T)**, which are DiI and phalloidin, and **(Q) **and **(S)**, which are DiI and TO-PRO-3. Panels labeled with an apostrophe (for example, **B',D'**) are single-channel images of DiI that correspond to the multichannel images with the same letter (for example, **B, D**). A subset of panels are cropped, digitally magnified, z-stacks of confocal images through the foregut **(B, F, J, N)**, prototroch **(D, H, L)**, brain **(P)**, forming ventral nerve cord **(Q)**, body ectoderm **(R) **and dorsal body ectoderm just posterior to the prototroch **(T)**. In **(B, F, J, N) **the foregut is outlined with a dashed line. The inset in **(D') **is an inverted, digitally magnified image of a single DiI-labeled prototroch cell with cilium (arrowhead). In **(P) **one brain lobe is outlined with a dashed line, and clusters of 2d descendants in the brain (arrow) are indicated. In **(P, Q) **2d descendants in the circumesophageal connectives (arrowheads) are indicated. In **(R') **the boundary between mesoderm and endoderm is indicated with a dashed line. In **(T) **the arrow points to unlabeled ectodermal cells (arrow). The blastomere labeled with DiI is indicated to the left of each row and the view is indicated in the lower-left corner of each panel (dors = dorsal, vent = ventral, llat = left lateral, rlat = right lateral). Anterior is to the left in all ventral, dorsal and left lateral images, and to the right in all right lateral images. The position of the mouth is indicated with an asterisk. ec = ectoderm, en = endoderm, ms = mesoderm, pt = prototroch, tt = telotroch, vn = ventral nerve cord.

**Figure 7:**
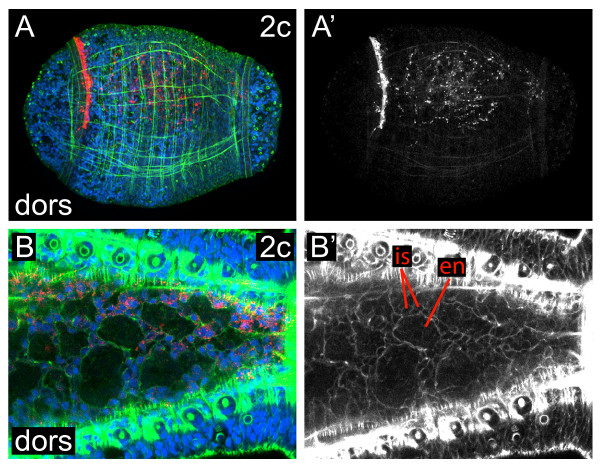
**Ectomesodermal descendants from 2c**. (**A, B**) Z-stacks of merged, confocal images from a dorsal (dors) view through a late stage 6 **(A) **or a stage 8 **(B) **larva after labeling micromere 2c. The channels are DiI (red), phalloidin (green) and TO-PRO-3 (blue). (**A'**) Single-channel image of DiI staining shown in **(A)**. **(B) **Cropped, digitally magnified image of the posterior trunk. (**B'**) Single-channel phalloidin image corresponding to the multichannel image in **(B)**. A large endodermal (en) cell and two smaller interstitial (is) cells are indicated. Anterior is to the left in all images.

Second quartet micromeres in *C. teleta *generate the fifth row of prototrochal cells (posterior supporting prototroch), trunk ectoderm, pygidial ectoderm, neurotroch, telotroch, pygidial ciliary band, foregut, ventral nerve cord ganglia, putative ectomesodermal cells and the rectum (Figure 6; Figure 7).

#### Micromere 2a

Descendants of 2a form the fifth row of prototrochal cells on the left side of the larva (Figure 5C,D; Figure 6A,D,D'). These cells have a single cilium (Figure 6D' inset, arrowhead). Micromere 2a also generates the majority of the left foregut, including the left presumptive pharynx and esophagus (Figure 6B,B',C) and a few cells in the left posterior mouth. In addition, 2a gives rise to cells positioned between the mesodermal bands and endoderm on the ventral and lateral sides of the larva, between the ectoderm and endoderm on the dorsal side of the larva, and scattered throughout the midgut (Figure 6C). These labeled cells have a mesenchymal shape and extend throughout the trunk and pygidium on the left side of the larva (similar to the pattern seen after labeling 2c) (Figure 7). During the stages analyzed, these cells had not yet adopted a terminally differentiated phenotype; therefore we do not know their ultimate fates. Based on their internal position and shape, these cells may be ectomesodermal. Descendants of 2a also contribute to the rectum (Figure 6c, arrow), which is discussed further in the 'Contributions to the hindgut' section below.

#### Micromere 2b

Micromere 2b gives rise to the medial-ventral region of the fifth row of prototrochal cells (Figure 5C, Figure 6E,H,H') and a small, medial-anterior region of the foregut and mouth (Figure 6F,F',G).

#### Micromere 2c

The pattern of labeled cells seen after injecting 2c is a mirror image of that after injecting 2a. Micromere 2c generates the right side of the fifth row of prototrochal cells (Figure 5C; Figure 6I,L,L'), the majority of the right foregut, including the right presumptive pharynx and esophagus (Figure 6J,J',K) and a few cells in the right posterior mouth. In addition, 2c gives rise to cells that are concentrated on the right side of the larva and are positioned between the mesodermal bands and endoderm on the ventral and lateral sides of the larva, between the ectoderm and endoderm on the dorsal side of the larva, and scattered throughout the midgut (Figure 6K; Figure 7). The 2a and 2c descendants in the midgut are distinct from the large, yolky endodermal cells (en) (Figure 7B,B'). These 2a/2c-derived interstitial cells (is) are smaller and are interspersed between the endodermal cells (Figure 7B,B'). Furthermore, these interstitial cells are concentrated towards the interface between the endoderm and mesodermal bands laterally or the endoderm and ectoderm dorsally. By contrast, endodermal cell nuclei are localized towards the interior of the gut and are not in the same plane as nuclei of the second quartet derivatives (Figure 7B). We hypothesize that these 2a/2c-derived interstitial cells may contribute to visceral mesoderm. Finally, descendants of 2c form part of the rectum (discussed below).

#### Micromere 2d

The 2d micromere is the largest micromere generated in *C. teleta*, and is born slightly before the other second quartet micromeres. Similar to many other annelids, in *C. teleta*, descendants of 2d generate the majority of ectoderm posterior to the prototroch (Figure 6M,O,Q,R,R',S). This includes both the segmented body ectoderm and the non-segmented pygidial ectoderm, which are separated by the telotroch (Figure 6M,O,S). One exception is a thin ring of 1d-derived cells that wrap around the trunk just posterior to the prototroch (Figure 6T,T', arrow). 2d also forms the neurotroch (Figure 6M), telotroch (tt) (Figure 6M,S) and pygidial ciliary bands. In addition, descendants of 2d form the ventral nerve cord (vn) (Figure 6Q), two small clusters of cells on the left-dorsal and right-dorsal sides of the brain (Figure 6P,P', arrow) and cells along the circumesophageal connectives (Figure 6P,P',Q, arrowheads). Although 2d does not contribute to the foregut (Figure 6N, N'; Figure 6O, dashed line), labeled surface ectodermal cells extend around the mouth opening (Figure 6M).

### Third quartet micromeres

**Figure 8:**
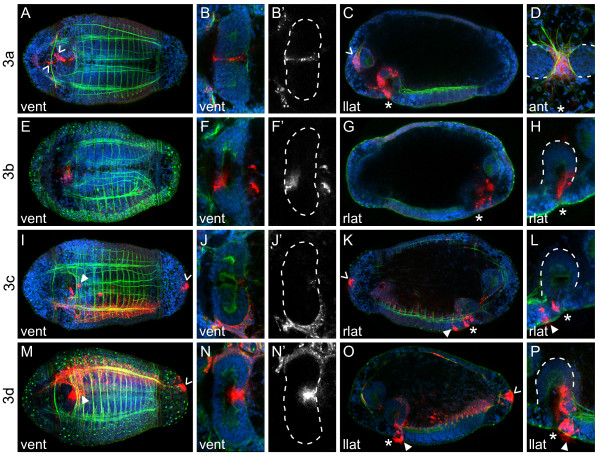
**Third quartet micromeres form the mesodermal bands and ectodermal structures**. (**A-P**) Z-stacks of merged, confocal images of late stage 6 larvae 5 days after labeling third quartet micromeres with DiI. The channels are DiI (red), phalloidin (green) and TO-PRO-3 (blue). Panels labeled with an apostrophe (for example, **B', F'**) are single-channel images of DiI corresponding to panels with the same letter (for example, **B, F**). Panels **(B, F, H, J, L, N, P) **are cropped, digitally magnified, z-stacks of confocal images through the foregut (dashed line). Panel **(D) **is a cropped, digitally magnified, z-stack of confocal images through anterior mesoderm, with brain demarcated (dashed line). Arrowheads point to DiI-labeled neurons in **(A)**, DiI-labeled anterior mesoderm in **(C)**, and DiI-labeled cells surrounding the anus **(I, K, M, O)**. Closed arrowheads point to DiI-labeled neurotroch cells in **(I, K, M, O)**. The blastomere labeled with DiI is indicated to the left of each row, and the view is indicated in the lower-left corner of each panel (dors = dorsal, vent = ventral, llat = left lateral, rlat = right lateral). Anterior is to the left in all ventral and left lateral images, to the right in all right lateral images and down in all anterior images. The position of the mouth is indicated with an asterisk.

The third quartet micromeres generate cells in the mouth, a small region of internal foregut, mesoderm in the head, and cells surrounding the anus. In addition, the third quartet micromeres form the left and right mesodermal bands, which give rise to most of the circular and longitudinal muscle fibers in the larva and the visceral mesoderm surrounding the foregut (Figure 8).

#### Micromere 3a

In *C. teleta*, micromere 3a generates cells on the left side of the mouth (Figure 8A,C) and a thin band of internally positioned medial foregut tissue (Figure 8B,B',C). By stage 8, some 3a descendants in the foregut contribute to a putative valve between the esophagus and midgut, which does not appear to be part of the foregut epithelium (not shown). Descendents of 3a also form a population of mesodermal cells in the head (Figure 8C,D), including a number of muscle cells with fibers extending posteriorly. These mesodermal cells are largely concentrated on the anterior side of the brain, with a few cells surrounding the lateral, ventral and posterior sides of the brain (Figure 8C, arrowhead; Figure 8D). Finally, 3a forms at least two neurons whose somas are positioned just anterior to the mouth on the ventral face of the animal (Figure 8A, arrowheads). Axons from these neurons extend along both sides of the ventral nerve cord.

#### Micromere 3b

Descendants of 3b give rise to cells on the right side of the mouth (Figure 8E) and a small, internally positioned, right-medial region of anterior foregut (Figure 8F-H). By stage 8, some 3b descendants in the foregut contribute to a putative valve between the esophagus and midgut (not shown). Because the 3b-derived and 3a-derived putative valve cells are in very similar positions between the foregut and midgut, we think it likely that they contribute to the same valve.

#### Micromere 3c

The 3c micromere forms the right mesodermal band (Figure 8I), which extends from just posterior of the telotroch anteriorly to the foregut. In general, the right mesodermal band forms both longitudinal and circular muscle fibers, and wraps around the entire right side of the foregut (Figure 8J.J'). Micromere 3c also generates a small number of cells in the right-posterior mouth (Figure 8I,K L), cells that surround the right side of the anus (Figure 8I,K, arrowhead), and a single, ciliated anterior neurotrochal cell on the ventral midline (Figure 8I,K,L, closed arrowhead).

#### Micromere 3d

The 3d micromere generates the left mesodermal band (Figure 8M), including longitudinal and circular body wall muscle fibers and mesoderm wrapping around the left foregut (Figure 8N,N'). 3d also gives rise to cells in the left-posterior mouth (Figure 8M,O,P), cells surrounding the left side of the anus (Figure 8M,O, arrowhead), and a few anterior ciliated neurotrochal cells (Figure 8M,O,P, closed arrowhead). In general, the 3d micromere forms a mirror-image clone to 3c, although 3d also generates a small, internally positioned, medial-posterior region of foregut (Figure 8N,N',P).

### Mesodermal band expansion

**Figure 9:**
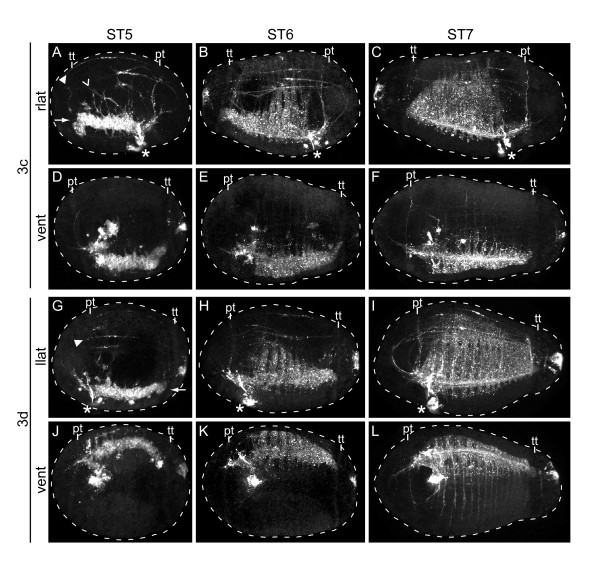
**Mesodermal band expansion**. (**A-L**) Single-channel, z-stacks of confocal images of larvae (dashed line) after labeling micromeres 3c or 3d with DiI (white). In **(A, G) **mesodermal bands (arrows), forming circular muscle fibers (arrowheads), and forming longitudinal muscle fibers (closed arrowheads) are indicated. The blastomere labeled with DiI is indicated to the left of each row, the stage of each larva is indicated above each column and the view is indicated to the left of each row (vent = ventral, llat = left lateral, rlat = right lateral). Anterior is to the left in all ventral and left lateral images and to the right in all right lateral images. The position of the mouth is indicated with an asterisk. The position of the prototroch (pt) and telotroch (tt) are indicated.

At stage 4, each mesodermal band is visible as a row of subsurface cells that extends from the telotroch anteriorly towards the foregut. At stage 5, the right and left mesodermal bands (Figure 9A, G, arrow), descendants of 3c and 3d, respectively, begin to extend muscle fibers (Figure 9A, D, G, J). At this stage, both longitudinal (Figure 9A,G, closed arrowhead) and circular (Figure 9A, arrowhead) muscle fibers can be seen, as well as muscle-cell soma that are positionally distinct from the mesodermal bands. These muscle cells originate from the mesodermal bands. Each mesodermal band is several cells wide along the dorsoventral and mediodistal axes, with the exception of the posterior end, which contains a single large cell. By stage 6, the mesodermal bands have begun expanding circumferentially in segmental rows. The first five segments extend dorsally (Figure 9B,H) but not ventrally (Figure 9E,K). By stage 7, most of the mesodermal segments have expanded both dorsally (Figure 9C,I) and ventrally (Figure 9F,L), and occupy the bulk of the larval body wall. The majority of the musculature in the head appears to arise from 3a, although thin muscle fibers, but not cell bodies, from 3c and 3d are visible in the head (Figure 9A-C,F,H,I,L). Previously, precursors of the larval body segments were described by Eisig in 1898 [14]. These structures were referred to as 'bauchplatten' or 'belly plates' and can be seen as a higher density of nuclei in the ventrolateral body of the larva, starting at stage 4. The belly plates expand dorsally and posteriorly as the larva develops [14,15,22]. Expansion of the mesodermal bands seen after labeling 3c and 3d corresponds to recent descriptions of belly plate expansion by Thamm and Seaver [22].

### 4d

#### Micromere 4d

**Figure 10:**
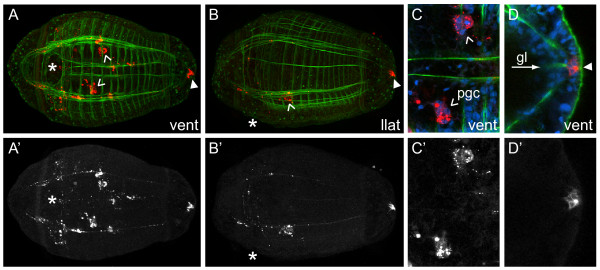
**Micromere 4d forms the primordial germ cells and anus**. (**A-D**) Z-stacks of merged, confocal images of late stage 6 larvae 5 days after labeling micromere 4d with DiI. The channels are DiI (red), phalloidin (green) and TO-PRO-3 (blue). Panels labeled with an apostrophe (for example, **A', B'**) are single-channel images of DiI that correspond to panels with the same letter (for example, **A, B**). Panels **(C, D) **are cropped, digitally magnified, z-stacks of confocal images showing the primordial germ cells (pgc) and anus, respectively. In **(A-C) **arrowheads point to primordial germ cells. In **(A, B, D) **a closed arrowhead points to DiI-labeled anal cells. In **(B) **the position of the mouth is marked with an asterisk. In **(D) **an arrow points to the gut lumen (gl). The view is indicated in the lower-right corner of each panel (vent = ventral, llat = left lateral). Anterior is to the left in all images.

When 4d is born, it is comparable in cell size to other third and fourth quartet micromeres. Concerning its fate, the 4d micromere generates a few longitudinal and circular body wall muscle fibers (Figure 10A-B'), although 3c and 3d form the majority of larval muscle fibers. Descendants of 4d are found at the posteriormost end of the gut, probably the presumptive anus (Figure 10A-B', D,D', closed arrowhead). These cells are positioned on the surface of the larva (Figure 10D, closed arrowhead) and contact the gut lumen (gl) (Figure 10D, arrow) at late stage 6. The 4d micromere also generates an internal pair of cell clusters that correspond to the presumptive primordial germ cells (pgc) (Figure 10A-C', arrowheads). These cells are in a similar position to nanos+, vasa+ and piwi+ cells at similar stages ([23] and Seaver laboratory, unpublished data). After labeling 4d, we also see spots of DiI in the endoderm. We do not think that that these DiI spots are labeled cells, because they do not appear to contain nuclei and they do not have a stereotypic position that remains constant from animal to animal. It is possible that some descendants of 4d undergo programmed cell death and that the remnants of these cells are visible in the endoderm. At this time, we do not have evidence that 4d contributes to midgut endoderm.

### Third and fourth quartet macromeres

Macromeres 3A, 3B, 3C and 4D generate the endoderm (Figure 2N-P, R), which eventually forms the intestinal midgut. In general, endoderm formed from 3A and 4D is restricted to the left side of the embryo, whereas endoderm from 3B and 3C is restricted to the right side of the embryo. Furthermore, endoderm descended from 3A and 3B is more anteriorly positioned whereas endoderm from 3C and 4D is more posteriorly positioned (Figure 2N-P, R), although the position of the clones within the gut varies from animal to animal.

### Contributions to the hindgut

#### Formation of the anus

**Figure 11:**
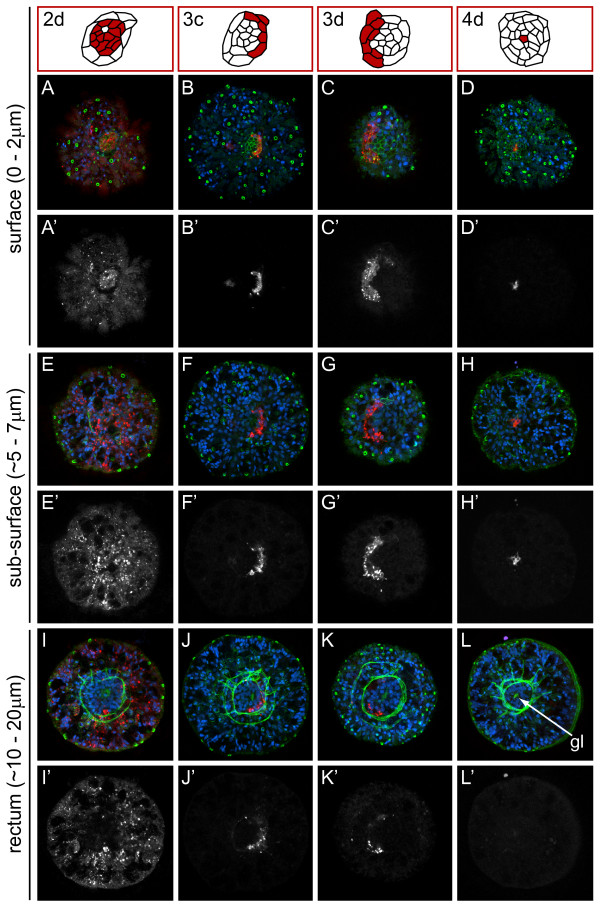
**Multiple micromeres contribute to the region surrounding the presumptive anus**. (**A-L**) Z-stacks of merged, confocal images of late stage 6 larvae 5 days after labeling micromeres 2d (column 1), 3c (column 2), 3d (column 3) or 4d (column 4) with DiI. The channels are DiI (red), phalloidin (green) and TO-PRO-3 (blue). Panels labeled with an apostrophe (for example, **A', B'**) are single-channel images of DiI that correspond to panels with the same letter (for example, **A, B**). The depths of the confocal z-stacks, starting at the surface **(A-D) **and progressing into the larva, are indicated to the left of each row. The diagrams at the top of each column are of the posterior rosette cells and are drawn from the images immediately below them (that is, surface z-stacks). In **(L) **an arrow points to the forming gut lumen (gl). All images are from a posterior view with ventral down.

Because several cells (2d, 3c, 3d and 4d) contribute to a small region at the posterior end of the larva, including the anus, we examined the relative position of these clones from a posterior view using confocal laser scanning microscopy (Figure 11). At late stage 6, there is a rosette (diagrams) of surface cells in the center of the pygidium (Figure 11A-D). The number of cells in the rosette increases from stage 5 to early stage 7. Immediately beneath this rosette, concentric rings of muscle fibers surround the rectum and possibly the posterior end of the midgut (Figure 11I-L). The forming gut lumen (gl) is also visible in the center of the rectum (Figure 10D, Figure 11L, arrow). 2d descendants form the majority of pygidial ectoderm. On the surface, 2d descendants form a large number of the rosette cells and cells surrounding the rosette (Figure 11, 2d diagram; Figure 11A, A', E, E'). At a deeper focal plane, 2d descendants can be seen in the pygidial ectoderm that surrounds the rectum (Figure 11I,I'). Descendants of micromere 3c form the right outer ring of rosette cells (Figure 11, 3c diagram; Figure 111B,B',F,F'). These cells extend below the surface and, at a deeper focal plane, abut the border between the muscle-fiber rings and the rectum (Figure 11J,J'). The posterior clone arising from micromere 3d is a mirror image of that generated by 3c (compare diagram 3d with 3c in Figure 11; Figure 11C,C',G,G',K,K'). The cells generated by 3c and 3d form some of the concentric rings of muscle fibers, although they appear to be only a subset of the fibers visible with phalloidin staining (not shown). We could not determine whether other micromeres also contribute to these muscle rings. Finally, 4d descendants form a few cells in the center of the rosette (Figure 11, 4d diagram; Figure 11D,D',H,H'). These 4d rosette cells extend from the surface towards the interior, where they contact the gut lumen (Figure 10D). We hypothesize that these 4d descendants form the future anus. 4d does not appear to generate any subsurface cells in the posterior end of the larva (Figure 11L,L').

#### Formation of the rectum

**Figure 12:**
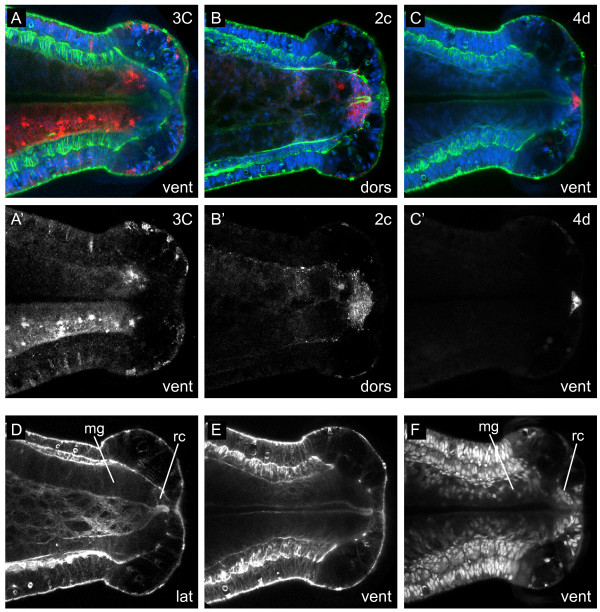
**Contributions to the developing gut at stage 8**. (**A-C**) Cropped z-stacks of merged, confocal images through the posterior end of stage 8 larvae at the level of the gut lumen 7 days after labeling 3C, 2c or 4d with DiI. The channels are DiI (red), phalloidin (green) and TO-PRO-3 (blue). The cell labeled with DiI is indicated in the upper-right corner. Panels labeled with an apostrophe (for example, **A', B'**) are single-channel images of DiI corresponding to panels with the same letter (for example, **A, B**). (**D-F**) Single-channel confocal z-stacks through the posterior end of stage 8 larvae labeled with phalloidin **(D, E) **or TO-PRO-3 **(F) **to show the intestinal lumen or cell nuclei, respectively. Panels **(E, F) **are corresponding single-channel images from **(C)**. The view is indicated in the lower-right corner of each panel (vent = ventral, lat = lateral). Anterior is to the left in all images. mg = midgut, rc = rectum.

Because the gut continues to form throughout larval development, we scored descendant clones at later stages (stage 8, 7 days after labeling) (Figure 12) to examine more closely the micromere contributions to the posterior end of the alimentary canal. We individually injected micromeres 2a, 2c, 3Q and 4d with DiI. At stage 8, 1 day before metamorphosis, regions within the posterior end of the gut are more readily discernible than at stage 6. At stage 8, a gut lumen can be seen extending from the posterior end of the esophagus to the anus, but not through it (Figure 12D-F). At this stage, the rectum is distinguishable from the midgut intestine by a few morphological traits. First, the nuclear organization and cell size of the rectal cells are distinct from the intestinal cells, which are larger and more loosely organized (Figure 12F). Second, the gut lumen narrows along the dorsoventral axis as it passes from the intestine into the rectum (Figure 12D). Third, the rectal portion of the gut lumen is more densely ciliated than the intestinal portion of the lumen when analyzed by anti-acetylated-tubulin staining (NPM, unpublished observations). We found that descendants of 3C and 3D form the posterior end of the intestine, but not the rectum (Figure 12A,A'). Descendants of 2a and 2c form the rectum (Figure 12B,B'), whereas descendants of 4d form the presumptive anus (Figure 12C,C'). Interestingly, the anal cells derived from 4d do not appear to change in shape or number (four cells) from stage 6 to stage 8 (compare Figure 10D,D' with Figure 12C,C').

## Discussion

### Axial relationships of the micromere quartets

**Figure 13:**
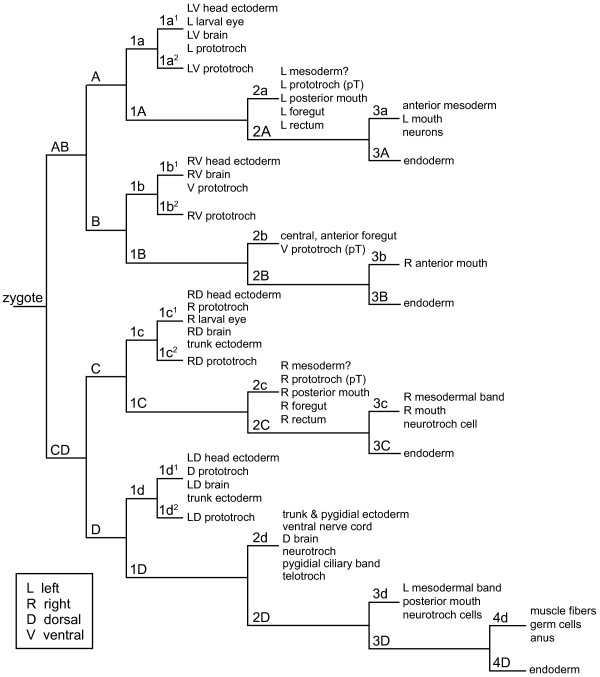
**Lineage tree showing blastomere fates in *Capitella teleta***. D = dorsal, L = left, pT = posterior supporting prototroch, R = right, V = ventral.

**Figure 14:**
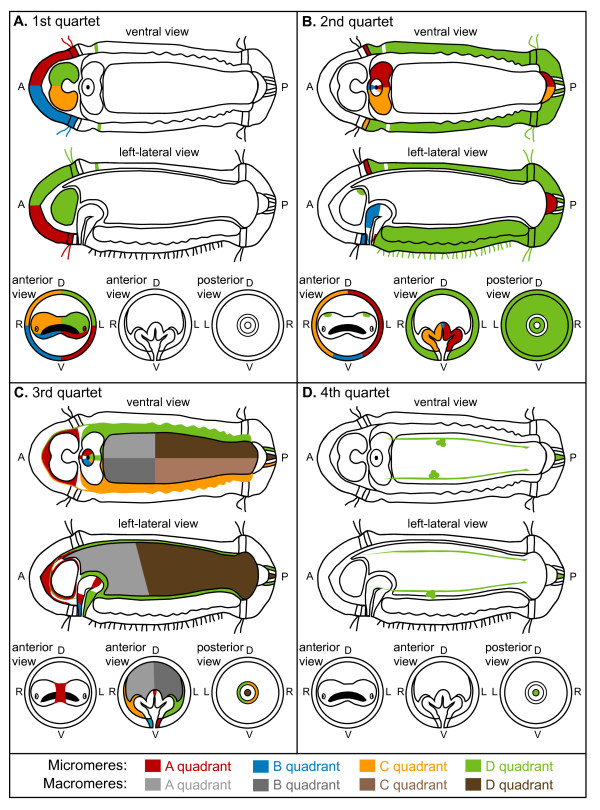
**Summary fate map diagrams for *Capitella teleta***. (**A-D**) Patterns seen after injecting DiI into individual micromeres or third quartet macromeres in *C. teleta*. Each panel corresponds to one micromere tier, and each color corresponds to one quadrant. The views are ventral, left lateral through the mouth, anterior through the brain, anterior through the foregut, and posterior through the forming anus. D = dorsal, L = left, R = right, V = ventral.

In spiralians for which a fate map exists, it has been noted that descendants of the micromere quartets are arranged similarly along the larval/adult body axes [6]. In the polyclad platyhelminth *Hoploplana inquilina*, the nemertean *Cerebratulus lacteus*, the mollusk *Patella vulgata*, and the annelids *C. teleta *and *P. dumerilii*, first quartet micromeres generate the left-ventral (1a), right-ventral (1b), right-dorsal (1c) and left-dorsal (1d) tissues. For the second and third quartet micromeres, the pattern seems less conserved. In *H. inquilina *and *C. lacteus*, second quartet micromeres are segregated into left (2a), right (2c), ventral (2b) and dorsal (2d) positions, whereas in *P. vulgata *and *C. teleta*, 2a and 2c clones are segregated into left and right positions, respectively. Descendants of third quartet micromeres are distributed similarly to those of the first quartet in *C. lacteus *and *P. vulgata *(Figure 13, Figure 14A-C) [12,24-26]. In some spiralians, the symmetry between sets of clones is more conserved than the position of the clones relative to the animal axes. For example, in *C. lacteus*, *P. vulgata*, *llyanassa obsoleta*, *Crepidula fornicata*, *C. teleta *and *Helobdella robusta*, 2a/2c and 3c/3d are mirror-image clones (Figure 14B, C) [10,25-28]. In the mollusks *P. vulgata*, *I. obseleta *and *C. fornicata*, and the nemertean *C. lacteus*, 3a/3b descendants also generate mirror-image clones [10,25,26,28]. In two annelids and a nemertean (*C. teleta*, *H. robusta*, *P. dumerilii *and *C. lacteus*), 1a/b and 1c/d generate mirror-image clones (Figure 14A) [12,26,27], whereas in mollusks (*Chaetopleura apiculata*, *I. obseleta*, *C. fornicata*), 1a/1c generate mirror-image clones [8,10,29]. Thus, although there are some similarities, the contributions relative to the plane of bilateral symmetry vary between micromere quartets within and across species.

### Origins of nervous systems

In annelids, the majority of anterior unsegmented ectoderm is generated by the 1q micromeres, whereas the majority of segmented trunk ectoderm is generated by the 2d micromere. Furthermore, the brain or supraesophageal ganglion comes from 1q (1q^1 ^where studied), and the ventral nerve cord comes from 2d in *Amphitrite ornata*, *Nereis*, *Scoloplos armiger*, *Helobdella *and *C. teleta *(Figure 13; Figure 14A,B) [12,27,30-34]. Within the context of the largely conserved origin of the annelid central nervous system from 1q and 2d, we find some unique features in *C. teleta*. Micromeres 1c^1 ^and 1d^1 ^generate the majority of the brain with only minor contributions from 1a^1 ^and 1b^1 ^(Figure 13; Figure 14A,B). Furthermore, a small population of 2d-derived cells are found in the brain and positioned along the circumesophageal connectives (Figure 6P,Q; Figure 14B). Second quartet contributions to the brain have not been reported in annelids. However, in the leech *H. robusta*, daughters of 2d (dnopq' and dnopq") form putative glial cells in the supraesophageal ganglion [27,35]. Based on their position, we think it is unlikely that the 2d-derived brain cells in *C. teleta *are glial.

There are several other cells that make minor contributions to the nervous system in the trunk of annelids. In *C. teleta*, 1c^1 ^and 1d^1 ^give rise to putative trunk sensory neurons, and 3a generates two neurons near the mouth. In *H. robusta*, micromeres 2a, 2c, 3c and 3d (a", c", c''', d''', respectively) are all reported to give rise to putative neurons and/or mesenchyme in the proboscis and the anterior sucker. Furthermore, 3a (a''') forms a pair of neuron-like cells in the subesophageal ganglion that project to the proboscis and posteriorly into the trunk [27]. This pair of neurons is similar in description to the pair of 3a-derived neurons by the mouth in *C. teleta *(Figure 8A, arrowheads). Unfortunately, we were unable to determine whether micromeres other than 1q, 2d and 3a generate neurons in *C. teleta*. Based on clonal position, we think it likely that the rows of peripheral neurons in the trunk are formed by 2d, and clusters of neurons in the foregut are derived from 2a and 2c. Taken together, these results suggest that the origin of the annelid nervous system from 1q and 2d is fairly conserved, although species-specific variations may emerge once more animals are studied in detail.

Direct comparisons of nervous system fate maps between annelids and other spiralians are more difficult. In gastropod mollusks, the ganglia of the central nervous system arise later in larval development [36-38], making fate mapping technically challenging. The nemertean *C. lacteus *undergoes a radical metamorphosis, with the larval nervous system being entirely replaced by derivatives of imaginal discs. The polyclad flatworm *H. inquilina *also has a biphasic life cycle, and the relationship of the larval nervous system to the adult nervous system is not clear. In the mollusks that have been examined, the anterior ectoderm is formed by descendants of the first quartet micromeres [8,10,25,29]. This suggests that the apical ganglion and the cerebral ganglia are generated by 1q micromeres, similar to annelids. There are some data that support this hypothesis. In *C. fornicata*, 1a, 1c and 1d contribute to the apical ganglion [10], and in *C. apiculata*, descendants of 1c^1 ^and 1d^1 ^form the apical organ [8]. A similar situation may exist in *C. lacteus*, as micromeres 1a and 1b were reported to give rise to the left and right larval cephalic discs [26]. In other nemerteans that develop via a pilidium larva (heteronemerteans), the cephalic discs are thought to generate the head ectoderm and cerebral ganglia of the juvenile [39-41].

Fate maps of ganglia in the body are more complicated to compare. In mollusks, the nemertean *C. lacteus*, and the polyclad flatworm *H. inquilina*, the 2d 'primary somatoblast' does not form the majority of body ectoderm as it does in annelids [8-10,21,24,26,28]], and contributions to ganglia in the body may also be more variable. In C. *fornicata*, 2b contributes to the supra-intestinal and sub-intestinal ganglia, and 2a and 2c contribute to the left and right pedal ganglia. Interestingly, 2d gives rise to the posterior mantle cell [10], which is one of the first neurons to differentiate, and its axons may serve as a scaffold for later central nervous system development [36,38]. Peripheral neurons in *C. fornicata *are generated by all 1q and 2q micromeres [10], whereas third quartet contributions to the nervous system have not yet been determined. In *C. lacteus *and *H. inquilina*, the first quartet forms the bulk of the larval ectoderm, and descendants of many first to third quartet micromeres contribute to the larval nervous system [24,26]. However, in heteronemerteans, the origin of the juvenile central nervous system in the trunk is not clear and awaits results from fate-mapping experiments. In contrast to annelids, in which 2d forms the majority of trunk ectoderm and all of the ganglia in the ventral nerve cord, multiple blastomeres may generate the central and peripheral nervous systems in the body of these other spiralians.

### Origins of ciliated bands

Patterns of ciliation (for example, ciliated 'bands') in marine larvae have played a central role in debates about the homology of larval structures and life history evolution across metazoan taxa [42-47]. The evolutionary significance of ciliary bands in metazoans remains unresolved. For example, some authors think that ciliated bands evolved as feeding structures associated with planktotrophy [48-53], whereas others argue that they are primarily locomotory in lecithotropic species [54-59]. Spiralians are a rich group of animals in which to explore these issues, as the pelagic phases of their life cycle bear many distinct ciliated structures (for example, akrotroch, prototroch, metatroch, telotroch and neurotroch), and the developmental origins of ciliated structures can be followed in detail due to the stereotyped spiral cleavage program.

Modern intracellular cell-lineage studies such as the work presented here give insights into some of these issues. The prototroch is found in a wide variety of spiralian groups (including polychaetes, echiurans, sipunculans, mollusks, nemerteans and entoprocts), and generally consists of multiple rows of cells bearing cilia. These larval cells are shed during metamorphosis and are often larger in size than adjacent cells because they become mitotically arrested during early cleavage stages. In general, the 1q^12 ^micromeres (accessory trochoblasts) contribute to the anteriormost prototrochal row (anterior supporting row), 1q^2 ^micromeres (primary trochoblasts) largely form the two most heavily ciliated prototrochal rows (main prototroch) and the 2q^11 ^micromeres (secondary trochoblasts) contribute to the posteriormost prototrochal row (posterior supporting row). There are variations in this pattern, and each tier of trochoblasts has been shown to contribute to multiple prototrochal rows. These generalizations are based on many early lineage studies (including [14,33,34,60-62] and on more recent intracellular fate mapping experiments in mollusks and a paleonemertean [10,28,29,63-65]. The same general pattern of contributions to each tier is found in *C. teleta *(Figure 5). To our knowledge, *C. teleta *is the only polychaete in which the precursors of all tiers of prototrochal precursors have been individually labeled. The clonal contribution to each row is less stereotyped than other studied spiralians, which is probably due to the relatively large number of cells in the prototroch. For example, in many mollusks, the entire prototroch can contain as few as 20 to 40 cleavage arrested cells [21], whereas the prototroch in *C. teleta *is composed of hundreds of smaller cells that interdigitate at clonal boundaries.

In spiralians, the D quadrant appears to have reduced its contribution to the prototroch relative to the other three quadrants. In most species studied, including *C. teleta*, the 2a, 2b and 2c, but not 2d micromeres make a contribution to the prototroch. The prototroch of the polychaetes *A. ornata *and *Podarke obscura *and the gastropod *C. fornicata *do not appear to have a contribution from 2d [10,34,62]. In *P. obscura*, descendents of 1a^1^, 1b^1 ^and 1c^1^, but not 1d^1 ^contribute to the prototroch [66]. In the chiton *C. apiculata*, the third quartet micromere, 3d, makes only a minor contribution to the prototroch [8]. In summary, the prototroch is widely found among spiralians, and the shared embryological origins of the prototroch between species provide evidence of it being a homologous structure.

Detailed information on the developmental origin of other trochal bands such as the metatroch, telotroch and neurotroch is much more limited. Intracellular fate-mapping studies of these other ciliated bands have not been performed on many species and they are not found on all spiralian taxa. For example, aplacophoran mollusk larvae have a telotroch [67], but gastropod mollusks and nemerteans do not have telotrochs or neurotrochs, although some mollusks have ciliated bands along the midline of the foot. Within annelids, not all polychaetes possess the same complement of ciliary bands. For example, *P. dumerilii *does not possess a neurotroch or telotroch. The neurotroch in the polychaete *S. armiger *is reported to be derived from the third quartet micromeres 3c and 3d [30]. In *C. teleta*, the first species in which intracellular lineage tracers were used to study the origin of a neurotroch, most of the neurotroch is generated by the 2d micromere (Figure 14B), whereas only small contributions near the mouth are provided by 3c and 3d (Figure 8, closed arrowheads). The telotroch and pygidial ciliary bands in *C. teleta *are generated entirely by derivatives of the 2d micromere (Figure 13, Figure 14B). The metatroch, the quintessential ciliary band required for 'opposed band feeding' in spiralian larvae, is not present in nemerteans nor in the majority of polychaete families, such as Capitellidae, which includes *C. teleta *[55,56]. The origin of the metatroch has only been studied in a single species of polychaete, *Polygordius*, in which it was reported to be derived from the third quartet micromeres 3c and 3d [68]. If true, then the metatroch of *Polygordius *is not developmentally homologous with the secondary ciliary band or the ciliated food grove of the molluscan velum, which are derived from 2a, 2b and 2c [10]. In another example, the third quartet micromeres 3a and 3b and derivatives of all first and second quartet micromeres give rise to the ciliated bands in the pilidium larva of the nemertean *C. lacteus *[26]. Thus, it appears that distinct blastomeres can be co-opted to form various ciliated bands in different spiralian lineages.

Nielsen suggests that prototrochs, metatrochs and telotrochs are all derived from a circumblastoporal ciliated band in a radially symmetrical, holopelagic ancestor, and that their differentiation was established in connection with the evolution of a new body axis, the anterior-posterior axis, forming at an angle to the primary, apical-blastoporal axis [46,52,69]. This is unlikely because most ciliated bands are not derived from the vicinity of the blastopore, which forms at the vegetal pole [70]. Furthermore, the multiple embryonic origins of ciliary bands imply that not all ciliary bands are homologous. The distinct embryological origins of ciliary bands and the multitude of ciliary band types in spiralians indicate that ciliary bands are evolutionarily labile, thus caution must be used in making statements about homology across distantly related taxa.

### Origins of mesoderm

The developmental origins of mesodermal cell types in *C. teleta *represent significant modifications of the typical spiralian fate map. One of the most important findings from spiralian cell-lineage studies is the documentation of dual embryological origins of mesoderm. One source is from ectomesoderm (cells that give rise to both ectodermal and mesodermal derivatives) and the other from endomesoderm ('embryonic precursors of both mesoderm and the endodermally derived intestine') [6,7]. Endomesoderm arises from a single fourth quartet micromere in the D quadrant, 4d, and its origin from 4d is a highly conserved feature within Spiralia (Table 2). In *C. teleta*, 4d generates mesodermal cell types typically found in other spiralians, including primordial germ cells and muscle cells, but it does not contribute to the endodermally derived intestine (Figure 13; Figure 14D). Although 4d makes a small contribution to the posterior gut in *C. teleta*, this contribution is to the anus, an ectodermal structure (Figure 10D). Therefore, we argue that in *C. teleta*, 4d is not a mesentoblast, but a mesectoblast. At first glance, it seems possible that 3D is the mesentoblast in *C. teleta*, because its descendants contribute to both the endodermal midgut and mesodermal structures. However, 3D also generates ectoderm (4d-derived anus), eliminating it as a mesentoblast candidate (Figure 14C). The descendants of 4d (DM'') in *Helobdella *are similar to those on *C. teleta*: 4d generates mesoderm and ectoderm, but not endoderm [32]. In the classic spiralian literature, there are other examples of 4d generating only mesoderm in polychaete annelids, including in *A. ornata *and *Arenicola cristata *[34,60]; however, these reports need to be confirmed by direct intracellular labeling experiments. It will be important to determine how widespread is the 4d restriction to mesodermal fates within Annelida.

**Table 2:** Embryological sources of mesoderm in spiralians^a,b^.

	Species	Ectomesoderm	Endomesoderm	Reference
Nemertean	*Cerebratulus*	3a, 3b	4d	[26]

Polyclad tubellarian	*Hoploplana*	2b	4d	[9,24]

	*Chaetopleura*	None	3D	[8]
	
Mollusk	*Patella*	3a, 3b	3D	[25]
	
	*Ilyanassa*	2b, 2c, 3d, 3A, 3B, 3C	4d	[28]
	
	*Crepidula*	2c, 3a, 3b	4d	[10]

Annelid	*Helobdella*	1a (a'), 1b (b'), 1c (c'), 1d (d'), 3a (a'''), 3c (c'''), 3d (dm') 4d (DM'')	None	[27,32]
	
	*Platynereis*	1A, 1B, 1C, 1D	4d	[12]
	
	*Capitella*	2a?, 2c?, 3a, 3b?, 3c, 3d, 4d	none	This report

^a^Examples included are taken from fate maps determined through use of intracellular lineage tracers injected into embryos at the 8-64 cell stage.

^b^Only blastomeres with confirmed mesoderm descendants are included.

In Wilson's initial account of *Nereis *development, all 4d derivatives were described to be mesodermal [33]. However, after Conklin's characterization of the *Crepidula *cell lineage [5], Wilson re-examined the early cleavages of 4d in *Nereis *and observed small daughter cells of 4d born before the formation of the mesodermal bands. These daughter cells contributed descendants to the posterior endodermal archenteron [7]. Wilson proposed an evolutionary scenario to explain the observed variation in the relative contribution of 4d to endoderm and mesoderm in spiralians [7]. He speculated that in the ancestral spiralian form, fourth quartet micromeres generated only endoderm. In subsequent spiralian descendants, mesodermal derivatives eventually segregated entirely to 4d. Over time, the capacity of 4d to form endoderm was reduced in annelids. From a current phylogenetic framework, it is difficult to find support for the idea that the spiralian ancestor lacked endomesoderm, because it is likely, based on comparative molecular and lineage studies, that endomesoderm was present in the bilaterian ancestor [71-74].

In contrast to the relatively conserved embryological origin of endomesoderm, the origin of ectomesoderm is more variable across spiralians (Table 2). In *C. teleta*, micromeres 3a, 3c, 3d, 4d and possibly 2a, 2c and 3b generate ectomesoderm (Figure 13, Figure 14B-D), and the contribution to ectomesoderm by second and third quartet micromeres is shared with mollusks, annelids and nemerteans. A notable exception is the generation of head mesoderm by first quartet micromeres in *H. robusta *(Table 2) [27]. Newby also described a contribution to mesoderm from first quartet micromeres in the echiuran *Urechis caupo *[75], although this has not been directly confirmed by intracellular lineage tracers. It is notable that the B quadrant does not generate mesoderm in *C. teleta*, with a minor possible exception of the 3b-derived valve between the esophagus and midgut, which may be mesodermal. B quadrant contributions to ectomesoderm are commonly found in spiralians, including in *C. lacteus*, *P. vulgata*, *I. obsoleta*, *C. fornicata*, *Helobdella *and *P. dumerilii*. In *H. inquilina*, 2b appears to be the only ectomesoblast (Table 2).

In addition to 4d not being a 'mesentoblast' in *C. teleta*, there is another modification of the typical 4d descendant fates. In most spiralians, 4d gives rise to the mesodermal bands, which extend along the anterioposterior axis and contribute to the trunk mesoderm [76]. In *C. teleta*, the mesodermal bands are generated by the third quartet micromeres 3c and 3d rather than by 4d (Figure 13; Figure 14C). In his classic study of *Capitella *development, Eisig also noted that 3c and 3d generate the coelomic linings [14]. Mesodermal bands arising from the 3c and 3d micromeres have not been previously reported for any other spiralian. Although generation of the mesodermal bands by 3c and 3d is unique to *Capitella *among the animals studied to date, other descendent cell types generated by 3c and 3d, such as contributions to the stomodeum, are shared with *P. vulgata*, *H. inquilina *and *C. lacteus *[24-26]. In addition, in *H. robusta *and probably in *P. dumerilii*, 3c and 3d generate mesoderm closely associated with the foregut [12,27], a pattern shared with *C. teleta*. Among the spiralians that have been examined by intracellular fate mapping experiments, contributions to visceral mesoderm by third quartet micromeres have not been reported outside annelids. In conclusion, modifications of the spiralian program are complex, and do not represent simple heterochronic shifts of fate or whole-cell fate transformations. In *C. teleta*, unlike in most other spiralians, the size of the 4d blastomere is not obviously larger than other fourth quartet micromeres, and the 3c and 3d micromeres are similar in size to the other third quartet micromeres. It is interesting to consider whether 4d acts as the organizer in *C. teleta*, as it does in some other spiralians [76], or whether this function is now assumed by cells that generate the mesodermal bands (3c and 3d).

In summary, the generation of the mesodermal bands by 3c and 3d in *C. teleta *represents a radical departure from the highly conserved 4d-derived mesodermal bands described in other spiralians. Furthermore, our lineage results indicate that although several distinct micromeres generate mesodermal derivatives in *C. teleta*, all of these arise from ectomesoderm, a feature shared with another annelid, *Helobdella*.

### Origins of the alimentary canal

#### Foregut

In spiralians, the anterior region of the gut is commonly derived from second (2q) and third quartet (3q) micromeres, although the contribution of particular blastomeres to mouth and foregut tissue varies among polychaete species, and between polychaete annelids and other spiralian taxa. Classic cell-lineage studies use the term 'stomatoblast' when referring to blastomeres that line the oral cavity and show that stomatoblasts are micromere derivatives of 2a to 2c in *Nereis*, 3a to 3d in *A. cristata*, 2a to 2c, 3a and 3b in *S. armiger*, and 2b, 3a and 3b in *P. obscura *[30,33,60,66]. These results are somewhat confusing because interpretations of particular cell fates are based on the author's definition of 'stomodeum' (stomodeum synonymous with pharynx [33], stomodeum synonymous with mouth [60]). Furthermore, some of these earlier studies inferred the fates of anterior gut cells based upon their positions during gastrulation. Some authors assumed that the mouth forms from the blastopore (*A. cristata *[60], *P. obscura *[66], *S. armiger *[30]), whereas others directly observed a distinct stomodeal opening at later stages (*Nereis *[33], *A. ornata *[34], *Capitella *[14]). Therefore, details from earlier fate maps of polychaetes should be interpreted and compared with caution.

In the fate map of *C. teleta *that we generated by intracellular injections, descendants of 2a to 2c and 3a to 3d contribute to cells in the mouth (Figure 13; Figure 14B,C). These results are generally consistent with results from classic lineage studies that examined micromere contributions to the stomodeum in other polychaete annelids. In *C. teleta*, the larval pharynx and esophagus show major contributions from 2a (left side) and 2c (right side) lineages, and a moderate contribution from 2b (central-anterior) (Figure 13; Figure 14B), which are similar in lineage and axial position to blastomeres traced by traditional methods in *Nereis*, *S. armiger *and probably *A. cristata *[30,33,60]. Taken together with *C. teleta*, these four polychaete species generate four distinct foregut architectures in the adult, but during developmental stages, the same micromeres form similar regions of the foregut. The larvae of these four species are predominantly lecithotrophic, and fate maps may be less conserved between these species and planktotrophic larvae that undergo substantial changes in larval tissues and organ systems during metamorphosis.

Modern fate maps have been published only for one other polychaete species, *P. dumerilii *[12]. In that study, 'stomodeum' broadly refers to the developing foregut, and contributions to the foregut fates were often inferred, because second or third quartet blastomeres were not injected directly. By injecting blastomeres at the eight-cell stage, Ackerman *et al*. showed that descendents of 1A and 1C, inferred to be 2a^2 ^and 2c^2^, contribute to the left and right stomodeum, respectively [12]. There was no apparent contribution to stomodeal tissue from 1B or 1D. This is in contrast to our findings in *C. teleta*, in which descendents of both B and D lineages could be traced to the mouth and/or foregut tissue (Figure 13; Figure 14B,C). Regarding oral ectoderm, Ackerman *et al. *[12] further corrected Wilson's [33] interpretations by stating that 3a to 3d are unlikely to be involved in 'the general ectoblast of the circum-oral and circum-anal regions.' This observation is notable because we show by direct injection that 3a to 3d generate ciliated ectodermal cells of the mouth in *C. teleta*. In the annelid *H. robusta*, Huang *et al*. found that all four first quartet micromeres contribute to the proboscis, along with 2a, 2c (a", c") and 3a and 3c (a''', c'''; discussed below) [27]. Second quartet micromeres having foregut-related fates are consistent with our findings in *C. teleta*; however, first quartet micromere contributions represent a significant departure from both modern and traditional cell-lineage studies in polychaetes.

Modern fate maps from molluscan and polyclad turbellarian embryos also show important differences from our map in *C. teleta*. For example, the respective foregut fates of second and third quartet micromeres in gastropod mollusks are essentially reversed in position relative to the 2q and 3q micromeres in polychaete annelids. In veliger larvae of both *C. fornicata *and *I. obsoleta*, 2q descendants give rise to the cells lining the mouth, and 3q descendants form the esophagus [10,28]. Within the trochophore larva of the limpet, *P. vulgata*, 2b, 3c and 3d all have internally positioned foregut fates [25]. In the polyclad *H. inquilina*, micromeres 2a, 2b and 2c contribute to oral ectoderm and associated cilia, whereas 3a, 3b and 3c were found to label oral cilia [24]. Only one third quartet micromere, 3d, makes a significant contribution to the mouth in *H. inquilina*. Although the relative positions of 2q and 3q descendants in the stomodeum of *H. inquilina *correlate with our results, one difference is that in *C. teleta*, 2a, 2b and 2c micromeres generate internal foregut tissues, unlike the pattern from any 2q labeling observed by Boyer *et al. *[24]. One generalization that can be made here is that micromeres from A and C quadrants in mollusk and polyclad embryos generate, respectively, the left and right structures of the larval mouth and foregut. For instance, Boyer *et al*. observed that in the blind gut of *H. inquilina*, 2a and 2c micromeres contribute to left and right regions of the stomodeum [24], which also applies to *C. teleta *and other polychaetes. However, there are important foregut-specific contributions in *C. teleta *that do not conform to any general patterns, at least until more spiralian taxa are examined by modern fate-mapping techniques.

#### Midgut

The midgut in *C. teleta *and its intestinal counterpart in other spiralians are derived entirely from endoderm. In most spiralian taxa for which cell-lineage data are available, including mollusks, annelids, nemerteans and sipunculans, 3A, 3B and 3C macromeres and all of their descendants generate midgut endoderm. Both descendents of 3D (4D, 4d) also generate endoderm in mollusk, nemertean and sipunculan embryos [10,26,28,77]. In polychaetes, 4D contributes to endoderm. 4d also contributes to endoderm tissue in some polychaete species [7,66], but not in others [12,30,34,60] including *C. teleta *(Figure 13; Figure 14D).

Similar to the axial map of micromere derivatives in both the foregut and nervous system, macromeres in *C. teleta *also contribute to the intestine in a quadrant-specific pattern. Descendants of 3A and 4D are located predominantly on the left side and those of 3B and 3C on the right side (Figure 14C). In *P. dumerilii*, 1A and 1C give rise to left and right midgut anlagen [12], which is consistent with a general pattern of quadrant-specific endoderm contributions in polychaetes. Our intracellular labeling also shows that the macromere contribution to anterior midgut in *C. teleta *makes a relatively abrupt junction with the esophagus, and therefore we conclude that there is no endodermal component of the foregut in this species. Furthermore, the macromere contribution to the intestine abuts the rectum at its posterior end, thus, endodermal cells do not generate hindgut tissue during larval development.

#### Hindgut

Compared with other gut territories, the posterior end of the alimentary canal or 'hindgut', has received little attention from previous cell-lineage studies on polychaetes. Traditionally the hindgut of polychaetes has been defined as a proctodeal invagination of ectoderm [18,19], implying that cells lining the rectal canal are internalized derivatives of surface ectoderm. However, in some spiralian cell-lineage studies, the hindgut has been described as endodermal [10,28]. For example, in gastropod mollusks, the posterior end of the gut or intestine is endodermal and derives from descendants of 4d [10,28,76]. The 4d-derived portion of the larval gut in *C. teleta *consists solely of a few surface cells restricted to the presumptive anus, which we interpret as ectodermal. Late in larval development, the gut canal passes through the posterior midgut and rectum, but not the presumptive anus. Based on this observation and our lineage data, we suggest that an anal opening is the last developmental step in forming a through gut in *C. teleta*, and that the hindgut (rectum plus anus) does not form by a proctodeal invagination of ectoderm. During metamorphosis the hindgut region probably undergoes structural modifications, and with further development, it becomes considerably longer than it was before the metamorphic transition from a non-feeding larva to a juvenile worm. We think it likely that the 2a/2c-derived rectum undergoes such structural modification.

I6*P. dumerilii*, Poonamali cellular contributions to the proctodeum have not been characterized [12], whereas in *Nereis*, Wilson mentions that 3q micromeres contribute to 'portions' of the circum-anal region [33]. Only Treadwell reports that specific micromere descendents of 2d^2^, 3c and 3d contribute to the proctodeal wall of *P. obscura *[66]. In *C. teleta*, we found that the rectum is derived from 2a and 2c, whereas the surrounding tissues are generated from 2d, 3c and 3d micromeres (Figure 14B, C), including a ring-like system of musculature that may function as a sphincter apparatus. The rectum abuts the 3C/4D-derived midgut endoderm on its anterior end and the 4d-derived presumptive anus on its posterior end (Figure 14C,D). Our map of this region establishes the most complete modern reference of posterior gut territories to date, and provides a framework that may be used to assess whether any generalized trends exist in other spiralians.

## Conclusions

Intracellular injection of the lineage tracer DiI into individual blastomeres (1q, 1q^1^, 1q^2^, 2q, 3q, 4d, 2Q, 3Q and 4D) in *C. teleta *has provided the most complete fate map for any polychaete annelid to date. Many features of the *C. teleta *fate map are similar to those found in other annelids, including the generation of the anterior ectoderm and prototroch by first quartet micromeres and the majority of post-trochal ectoderm by the primary somatoblast 2d. Increased resolution through the use of confocal laser scanning microscopy enabled us to generate a detailed fate map for regions such as the prototroch, mesoderm, nervous system and gut. Our analyses reveal complex contributions to these structures from several blastomere lineages. For example, more cells contribute to the posterior gut than previously documented for any other spiralian and four to seven distinct blastomeres generate mesoderm in *C. teleta*, all from ectomesodermal sources. We also identified modifications of the typical spiralian fate map in *C. teleta*; the most dramatic difference is the formation of the mesodermal bands by 3c and 3d rather than by 4d, which generates the mesodermal bands in all other spiralians examined. The fate map of *C. teleta *will provide a framework for future comparisons with other spiralian fate maps and the foundation for functional experiments of cell-fate determination in this species.

## Methods

### Animal care

A laboratory colony of *C. teleta *was maintained and embryos were collected as described previously [15], with the exception that adult animals were kept in filtered seawater (FSW) (passed through 20 μm filter) at 19°C. After injection, embryos were raised at 19°C in FSW (through 0.2 μm filter) with 60 μg/ml penicillin (Sigma-Aldrich Co., St Louis, MO, USA) and 50 μg/ml streptomycin (Sigma-Aldrich Co.) added.

### DiI labeling

For injections, the egg shell was permeabilized by a 30-second incubation in a 1:1 mixture of fresh 1 mol/L sucrose and 0.25 mol/L sodium citrate followed by two to three rinses with FSW. Individual blastomeres were pressure-injected with 1 part DiIC_18_(3) (1,1'-dioctadecyl-3,3,3',3'-tetramethylindocarbocyanine perchlorate; Invitrogen, Carlsbad, CA, USA) saturated in ethanol to 19 parts soybean oil (Wesson; ConAgra Foods Inc., Omaha, NE, USA) using aluminosilicate needles (Sutter Instrument Co., Novata, CA, USA). After labeling, uninjected and injected animals from the same brood (same stage of development) were raised for the same amount of time, and rates of development and morphological features were compared. An experiment was not scored unless 90% of the injected and 90% of the uninjected animals were healthy (morphologically normal and similar timing of development).

Animals at various developmental stages were imaged either live or fixed (DiI fixation) [78]. For compound fluorescence microscopy, fixed animals were incubated overnight at 4°C in a fluorescently conjugated toxin that recognizes F-actin (BODIPY FL-phallacidin; Invitrogen) diluted 1:100 in phosphate-buffered saline (PBS), then rinsed three to four times in PBS, incubated in 80% glycerol in PBS plus 0.125 μg/mL bisbenzimide Hoechst 33342 (Sigma-Aldrich Co.) for 3 to 12 h at 4°C and then analyzed. For confocal laser scanning microscopy, animals were incubated overnight at 4°C in a 1:1000 dilution of a nucleic acid stain (TO-PRO-3 iodide; Invitrogen) and 1:100 dilution of a phallotoxin (either BODIPY FL-phallacidin or Alexa Fluor 488-phalloidin; Invitrogen) in PBS, rinsed three to four times in PBS, then incubated in mounting media with an antifade reagent (SlowFade Gold; Invitrogen) for at least 3 h at 4°C, and analyzed.

### Microscopy

For compound fluorescence imaging, live and fixed DiI-labeled animals were imaged (Axioskop 2 Plus with an AxioCam HRm camera; both Carl Zeiss Inc., Munich, Germany) and analyzed (Openlab software, version 4.0.1; PerkinElmer Inc., Waltham, MA, USA). Confocal imaging was performed using a confocal microscope (LSM 510; Carl Zeiss). Z-stack projections were then generated (ImageJ; National Institutes of Health, Bethesda, MD, USA). Figures were constructed using illustration software (Photoshop CS4 and Illustrator CS4; Adobe Systems Inc., San Jose, CA, USA).

## Competing interests

The authors declare that they have no competing interests.

## Authors' contributions

NPM carried out the injections, animal care, data collection and analyses, and illustration preparation. MJB and ECS helped with initial data analyses, and MJB contributed to illustration preparation. MQM contributed to initial injections. All authors contributed to data interpretation and writing and editing of the manuscript. All authors read and approved this manuscript.

## References

[B1] Dunn CW Hejnol A Matus DQ Pang K Browne WE Smith SA Seaver E Rouse GW Obst M Edgecombe GD Sørensen MV Haddock SH Schmidt-Rhaesa A Okusu A Kristensen RM Wheeler WC Martindale MQ Giribet G Broad phylogenomic sampling improves resolution of the animal tree of life Nature 2008 452 745 749 10.1038/nature06614 18322464

[B2] Giribet G Dunn CW Edgecombe GD Hejnol A Martindale MQ Rouse GW Telford MJaDTJL Assembling the spiralian tree of life Animal Evolution: Genomes, Fossils, and Trees 2009 Oxford: Oxford University Press 52 64

[B3] Hejnol A Obst M Stamatakis A Ott M Rouse GW Edgecombe GD Martinez P Baguna J Bailly X Jondelius U Wiens M Müller WE Seaver E Wheeler WC Martindale MQ Giribet G Dunn CW Assessing the root of bilaterian animals with scalable phylogenomic methods Proc Biol Sci 2009 276 4261 4270 10.1098/rspb.2009.0896 19759036 PMC2817096

[B4] Helmkampf M Bruchhaus I Hausdorf B Multigene analysis of lophophorate and chaetognath phylogenetic relationships Mol Phylogenet Evol 2008 46 206 214 10.1016/j.ympev.2007.09.004 17937996

[B5] Conklin EG The embryology of *Crepidula* J Morphol 1897 13 1 226 10.1002/jmor.1050130102

[B6] Henry JJ Martindale MQ Conservation and innovation in spiralian development Hydrobiologia 1999 402 255 265 10.1023/A:1003756912738

[B7] Wilson EB Considerations on cell-lineage and ancestral reminiscence Ann NY Acad Sci 1898 XI 1 27 10.1111/j.1749-6632.1898.tb54960.x

[B8] Henry JQ Okusu A Martindale MQ The cell lineage of the polyplacophoran, *Chaetopleura apiculata*: variation in the spiralian program and implications for molluscan evolution Dev Biol 2004 272 145 160 10.1016/j.ydbio.2004.04.027 15242797

[B9] Boyer BC Henry JQ Martindale MQ Dual origins of mesoderm in a basal spiralian: cell lineage analyses in the polyclad turbellarian *Hoploplana inquilina* Dev Biol 1996 179 329 338 10.1006/dbio.1996.0264 8903349

[B10] Hejnol A Martindale MQ Henry JQ High-resolution fate map of the snail *Crepidula fornicata*: the origins of ciliary bands, nervous system, and muscular elements Dev Biol 2007 305 63 76 10.1016/j.ydbio.2007.01.044 17346693

[B11] Henry JJ Martindale MQ The origins of mesoderm in the equal-cleaving nemertean worm *Cerebratulus lacteus* Biological Bulletin 1996 191 286 288 10.2307/1542927 29220266

[B12] Ackermann C Dorresteijn A Fischer A Clonal domains in postlarval *Platynereis dumerilii *(Annelida: Polychaeta) J Morphol 2005 266 258 280 10.1002/jmor.10375 16170805

[B13] Blake JA Grassle JP Eckelbarger KJ *Capitella teleta*, a new species designation for the opportunistic and experimental *Capitella *sp. I, with a review of the literature for confirmed records Zoosymposia 2009 2 25 53

[B14] Eisig H Zur Entwicklungsgeschichte der Capitelliden Mittheilungen Aus der Zoologischen Station Zu Neapel 1898 13 1 292

[B15] Seaver EC Thamm K Hill SD Growth patterns during segmentation in the two polychaete annelids, *Capitella *sp. I and *Hydroides elegans*: comparisons at distinct life history stages Evol Dev 2005 7 312 326 10.1111/j.1525-142X.2005.05037.x 15982368

[B16] Eckelbarger KJ Grassle JP Interspecific variation in genital spine, sperm, and larval morphology in six sibling species of *Capitella* Bull Biol Soc Wash 1987 7 62 76

[B17] Boyle MJ Seaver EC Developmental expression of foxA and gata genes during gut formation in the polychaete annelid, *Capitella *sp. I Evol Dev 2008 10 89 105 10.1111/j.1525-142X.2007.00216.x 18184360

[B18] Michel C Westheide W, Hermans CO Intestine and digestive glands The Ultrastructure of Polychaeta (Microfauna Marina) 1988 4 5 175

[B19] Tzetlin A Purshke G Pharynx and intestine Hydrobiologia 2005 535/536 199 225 10.1007/s10750-004-1431-z

[B20] Rhode B Larval and adult eyes in *Capitella spec. I *(Annelida, Polychaeta) J Morphol 1993 217 327 335 10.1002/jmor.1052170307 29865472

[B21] Damen P Cell-lineage, and specification of developmental fate and dorsoventral organisation in the mollusc *Patella vulgata* Thesis, Univ Utrecht 1994

[B22] Thamm K Seaver EC Notch signaling during larval and juvenile development in the polychaete annelid *Capitella *sp. I Dev Biol 2008 320 304 318 10.1016/j.ydbio.2008.04.015 18511030

[B23] Dill KK Seaver EC Vasa and nanos are coexpressed in somatic and germ line tissue from early embryonic cleavage stages through adulthood in the polychaete *Capitella *sp. I Dev Genes Evol 2008 218 453 463 10.1007/s00427-008-0236-x 18651171

[B24] Boyer BC Henry JJ Martindale MQ The cell lineage of a polyclad turbellarian embryo reveals close similarity to coelomate spiralians Dev Biol 1998 204 111 123 10.1006/dbio.1998.9084 9851846

[B25] Dictus WJ Damen P Cell-lineage and clonal-contribution map of the trochophore larva of *Patella vulgata *(mollusca) Mech Dev 1997 62 213 226 10.1016/S0925-4773(97)00666-7 9152012

[B26] Henry JJ Martindale MQ Conservation of the spiralian developmental program: cell lineage of the nemertean, *Cerebratulus lacteus* Dev Biol 1998 201 253 269 10.1006/dbio.1998.8966 9740663

[B27] Huang FZ Kang D Ramirez-Weber FA Bissen ST Weisblat DA Micromere lineages in the glossiphoniid leech *Helobdella* Development 2002 129 719 732 11830572 10.1242/dev.129.3.719

[B28] Render J Cell fate maps in the *Ilyanassa obsoleta *embryo beyond the third division Dev Biol 1997 189 301 310 10.1006/dbio.1997.8654 9299122

[B29] Render J Fate maps of the first quartet micromeres in the gastropod *Ilyanassa obsoleta* Development 1991 113 495 501 1782861 10.1242/dev.113.2.495

[B30] Anderson DT The embryology of the polychaete *Scoloplos armiger* Q J Microsc Sci 1959 100 89 166

[B31] Nardelli-Haefliger D Shankland M Lox10, a member of the NK-2 homeobox gene class, is expressed in a segmental pattern in the endoderm and in the cephalic nervous system of the leech *Helobdella* Development 1993 118 877 892 7915671 10.1242/dev.118.3.877

[B32] Weisblat DA Kim SY Stent GS Embryonic origins of cells in the leech *Helobdella triserialis* Dev Biol 1984 104 65 85 10.1016/0012-1606(84)90037-X 6734941

[B33] Wilson EB The cell-lineage of *Nereis*. A contribution to the cytogeny of the annelid body J Morphol 1892 6 361 480 10.1002/jmor.1050060301

[B34] Mead AD The early development of marine annelids J Morphol 1897 XIII 227 327 10.1002/jmor.1050130202

[B35] Dohle W The ancestral cleavage pattern of the clitellates and its phylogenetic deviations Hydrobiologia 1999 402 267 283 10.1023/A:1003709129576

[B36] Croll RP Developing nervous systems in molluscs: navigating the twists and turns of a complex life cycle Brain Behav Evol 2009 74 164 176 10.1159/000258664 20029181

[B37] Dickinson AJ Croll RP Development of the larval nervous system of the gastropod *Ilyanassa obsoleta* J Comp Neurol 2003 466 197 218 10.1002/cne.10863 14528448

[B38] Dickinson AJ Nason J Croll RP Histochemical localization of FMRFamide, serotonin and catecholamines in embryonic *Crepidula fornicata *(Gastropoda, Prosobranchia) Zoomorphology 1999 119 49 62 10.1007/s004350050080

[B39] Bürger O Studien zu einer Revision der Entwicklungsgeschichte der Nemertinen 1894 Ber Naturf Ges Freiburg

[B40] Maslakova SA The Invention of the Pilidium Larva in an Otherwise Perfectly Good Spiralian Phylum Nemertea Integr Comp Biol 2010 10.1093/icb/icq096 21558236

[B41] Salensky W Morphogenetische Studien an Wurmern. II. Uber die Morphogenese der Nemertinen. Entwiklungsgeschichte der Nemertine im Inneren des Pilidiums Mem Acad Sci St Petersb 1912 30 1 74

[B42] Arendt D Technau U Wittbrodt J Evolution of the bilaterian larval foregut Nature 2001 409 81 85 10.1038/35051075 11343117

[B43] Garstang W The morphology of the Tunicata and its bearing on the phylogeny of the Chordata Q J Microsc Sci 1928 72 51 54

[B44] Hatschek B Studien über die Entwicklungsgsgeschichte der Anneliden. Ein Beitrag zur Morphologie der Bilaterien Arb Zool Inst Univ Wien 1878 1 277 404

[B45] Lacalli TC Ciliary bands in echinoderm larvae: evidence for structural homologies and a common plan Acta Zool 1993 74 127 133 10.1111/j.1463-6395.1993.tb01229.x

[B46] Nielsen C Nørrevang A Conway Morris S, George JD, Gibson R, Platt HM The Trochaea theory: an example of life cycle phylogeny The Origins and Relationships of Lower Invertebrates 1985 Oxford: Clarendon Press 28 41

[B47] Rieger RM The biphasic life cycle--a central theme of metazoan evolution Am Zool 1994 34 484 491

[B48] Jägersten G Further remarks on the early phylogeny of Metazoa Zool Bider Upps 1959 33 79 108

[B49] Jägersten G Evolution of the Metazoan life cycle 1972 London: Academic Press

[B50] Nielsen C Larval ciliary bands and metazoan phylogeny Fortschr Zool Syst Evolutionsforsch 1979 1 178 184

[B51] Nielsen C Origin and evolution of animal life cycles Biol Rev 1998 73 125 155 10.1017/S0006323197005136

[B52] Nielsen C How did indirect development with planktotrophic larvae evolve? Biol Bull 2009 216 203 215 19556589 10.1086/BBLv216n3p203

[B53] Strathmann RR The evolution and loss of feeding larval stages of marine invertebrates Evolution 1978 32 894 906 10.2307/2407502 28567936

[B54] Degnan SM Degnan BM The origin of the pelagobenthic metazoan life cycle: what's sex got to do with it? Integr Comp Biol 2006 46 683 690 10.1093/icb/icl028 21672778

[B55] Rouse GW Trochophore concepts: ciliary bands and the evolution of larvae in spiralian Metazoa Biol J Linn Soc 1999 66 411 464 10.1111/j.1095-8312.1999.tb01920.x

[B56] Rouse GW The epitome of hand waving? Larval feeding and hypotheses of metazoan phylogeny Evol Dev 2000 2 222 233 10.1046/j.1525-142x.2000.00063.x 11252565

[B57] Runnegar B No evidence for planktotrophy in Cambrian molluscs Evol Dev 2007 9 311 312 10.1111/j.1525-142X.2007.00165.x 17651352

[B58] Salvini-Plawen LV Was ist eine Trochophora? Eine Analyse der Larventypen mariner Protostomier Zool Jb Anat 1980 103 389 423

[B59] Sly BJ Snoke MS Raff RA Who came first--larvae or adults? origins of bilaterian metazoan larvae Int J Dev Biol 2003 47 623 632 14756338

[B60] Child CM The early development of *Arenicola *and *Sternaspis* Arch EntwMech Org 1900 9 587 723

[B61] Heath H The development of Ischnochiton Zool Jahrb, Abt Anat Ontog Tiere 1899 12 567 656

[B62] Treadwell AL The cell lineage of *Podarke obscura* Zool Bull 1897 1 195 203 10.2307/1535429

[B63] Damen P Dictus WJ Cell lineage of the prototroch of *Patella vulgata *(Gastropoda, Mollusca) Dev Biol 1994 162 364 383 10.1006/dbio.1994.1094 8150201

[B64] Maslakova SA Martindale MQ Norenburg JL Fundamental properties of the spiralian developmental program are displayed by the basal nemertean *Carinoma tremaphoros *(Palaeonemertea, Nemertea) Dev Biol 2004 267 342 360 10.1016/j.ydbio.2003.10.022 15013798

[B65] Maslakova SA Martindale MQ Norenburg JL Vestigial prototroch in a basal nemertean, *Carinoma tremaphoros *(Nemertea; Palaeonemertea) Evol Dev 2004 6 219 226 10.1111/j.1525-142X.2004.04027.x 15230962

[B66] Treadwell AL Cytogeny of *Podarke obscura *Verrill J Morphol 1901 17 399 486 10.1002/jmor.1050170304

[B67] Okusu A Embryogenesis and development of *Epimenia babai *(Mollusca Neomeniomorpha) Biol Bull 2002 203 87 103 10.2307/1543461 12200259

[B68] Woltereck R Beiträge zur praktischen Analyse der *Polygordius*-Entwicklung nach dem "Nordsee-" und dem "Mittelmeer-Typus" Arch EntwMech Org 1904 18 377 403

[B69] Nielsen C Six major steps in animal evolution: are we derived sponge larvae? Evol Dev 2008 10 241 257 10.1111/j.1525-142X.2008.00231.x 18315817

[B70] Martindale MQ Hejnol A A developmental perspective: changes in the position of the blastopore during bilaterian evolution Dev Cell 2009 17 162 174 10.1016/j.devcel.2009.07.024 19686678

[B71] Henry JQ Martindale MQ Boyer BC The unique developmental program of the acoel flatworm, *Neochildia fusca* Dev Biol 2000 220 285 295 10.1006/dbio.2000.9628 10753516

[B72] Martindale MQ Henry JQ Intracellular fate mapping in a basal metazoan, the ctenophore *Mnemiopsis leidyi*, reveals the origins of mesoderm and the existence of indeterminate cell lineages Dev Biol 1999 214 243 257 10.1006/dbio.1999.9427 10525332

[B73] Martindale MQ Pang K Finnerty JR Investigating the origins of triploblasty: 'mesodermal' gene expression in a diploblastic animal, the sea anemone *Nematostella vectensis *(phylum, Cnidaria; class, Anthozoa) Development 2004 131 2463 2474 10.1242/dev.01119 15128674

[B74] Technau U Scholz CB Origin and evolution of endoderm and mesoderm Int J Dev Biol 2003 47 531 539 14756329

[B75] Newby WW The Embryology of the Echiuroid Worm Urechis caupo 1940 Philadelphia: The American Philosophical Society

[B76] Lambert JD Mesoderm in spiralians: the organizer and the 4d cell J Exp Zool B Mol Dev Evol 2008 310 15 23 10.1002/jez.b.21176 17577229

[B77] Torrey JC The early embryology of *Thalassema mellita *(Conn) Ann NY Acad Sci 1903 14 165 246 10.1111/j.1749-6632.1901.tb55053.x

[B78] Meyer NP Seaver EC Neurogenesis in an annelid: characterization of brain neural precursors in the polychaete *Capitella *sp. I Dev Biol 2009 335 237 252 10.1016/j.ydbio.2009.06.017 19540831

